# Novel Methods and Approaches for Safety Evaluation of Nanoparticle Formulations: A Focus Towards *In Vitro* Models and Adverse Outcome Pathways

**DOI:** 10.3389/fphar.2021.612659

**Published:** 2021-09-09

**Authors:** Mounika Gayathri Tirumala, Pratibha Anchi, Susmitha Raja, Mahesh Rachamalla, Chandraiah Godugu

**Affiliations:** ^1^ Department of Regulatory Toxicology, National Institute of Pharmaceutical Education and Research (NIPER), Hyderabad, India; ^2^ Department of Biology, University of Saskatchewan, Saskatoon, SK, Canada

**Keywords:** nanotoxicity assessment, nanoparticles, nanomedicine, nanotechnology, *in vitro* assay

## Abstract

Nanotoxicology is an emerging field employed in the assessment of unintentional hazardous effects produced by nanoparticles (NPs) impacting human health and the environment. The nanotoxicity affects the range between induction of cellular stress and cytotoxicity. The reasons so far reported for these toxicological effects are due to their variable sizes with high surface areas, shape, charge, and physicochemical properties, which upon interaction with the biological components may influence their functioning and result in adverse outcomes (AO). Thus, understanding the risk produced by these materials now is an important safety concern for the development of nanotechnology and nanomedicine. Since the time nanotoxicology has evolved, the methods employed have been majorly relied on *in vitro* cell-based evaluations, while these simple methods may not predict the complexity involved in preclinical and clinical conditions concerning pharmacokinetics, organ toxicity, and toxicities evidenced through multiple cellular levels. The safety profiles of nanoscale nanomaterials and nanoformulations in the delivery of drugs and therapeutic applications are of considerable concern. In addition, the safety assessment for new nanomedicine formulas lacks regulatory standards. Though the *in vivo* studies are greatly needed, the end parameters used for risk assessment are not predicting the possible toxic effects produced by various nanoformulations. On the other side, due to increased restrictions on animal usage and demand for the need for high-throughput assays, there is a need for developing and exploring novel methods to evaluate NPs safety concerns. The progress made in molecular biology and the availability of several modern techniques may offer novel and innovative methods to evaluate the toxicological behavior of different NPs by using single cells, cell population, and whole organisms. This review highlights the recent novel methods developed for the evaluation of the safety impacts of NPs and attempts to solve the problems that come with risk assessment. The relevance of investigating adverse outcome pathways (AOPs) in nanotoxicology has been stressed in particular.

## Introduction

Nanoscience is an interdisciplinary area that utilizes thousand millionths of a meter (10^–9^ m) in size at least in one dimension for manipulating the properties different from the bulk material of the same chemical compound (Boisseau et al., 2007; Bayda et al., 2020). Although nanoscience has progressed over the course of decades, the use of nanotechnology became clear in the late 20th century (Hochella, 2002; Valcárcel et al., 2008; Schaming and Remita, 2015). Nanotechnology is an integrative approach that provides tools and technologies to study, modify, and control the applications of nanoscience with the fusion of multidisciplinary areas (Porter and Youtie, 2009; Saez et al., 2010). Despite its small size, nanotechnology has emerged in multiple fields with invulnerable progress and today its revolution in the world can be observed from the simple example of first-generation-tabletop televisions to the wall-hanging LED televisions, while in the biological areas, it aided in studying the interactions and behavior of biomolecules of cells due to which targeted-based approaches have become the first line of therapy nowadays (Teixeira et al., 2020). Thus, it can be observed that nanotechnology impacted our daily life with its tremendous contributions in the fields of electrics, electronics, medicine, engineering, artificial intelligence, etc., with the manipulation of nanometer-scale materials into a wide variety of innovations (Mahbub and Hoque, 2020), while the lessons of COVID-19 also taught us that we need to be quick and flexible with tools to handle the situation at any need of the hour in either diagnosis or treatment. Advances in nanotechnology will revolt against such pandemic situations in the future if oriented towards that direction.

As nanotechnology is seen around the neck today, materials that form the core of the nanotechnology and exhibiting size-dependent activities have gained immense importance with their diverse utilities. Initially, almost 90% of nanomaterials (NMs) were made from silicon dioxide (SiO_2_), carbon black, silver (Ag), and titanium dioxide (TiO_2_), which were gradually replaced by fullerenes, carbon nanotubes (CNTs), graphene, nanocellulose, polymers, nanofibrils, dendrimers, etc. (Khataee and Mansoori, 2011; Choubey et al., 2013; Ruiz-Palomero et al., 2017; Jebali et al., 2018). When NMs entered medical applications, they transformed the traditional procedures which are now found effective towards treating simple to complex disease conditions. For instance, flexible quantum dots and gold nanoparticles (NPs) are employed in the diagnosis of single base mismatch DNA detection, imaging, and molecular labeling (Coto-García et al., 2011). With ever-increasing applications, nanomedicine remains to be a steadily growing interdisciplinary field and shifted the paradigm in the medical world with rapid developments including diagnosis, monitoring, and treatment procedures with fewer adverse outcomes (AO) (Fang and Zhang, 2010; Tinkle et al., 2014). The actual progress of any research is observed with the clinical translation and that scenario can be witnessed today with the availability of more than 50 nanoformulations in the global market which boomed the market with raise to $138.8 billion in 2016 from $53 billion in 2009, whereas anticancer drug applications are contributing major part (Shukla et al., 2020). In 2019, paclitaxel-enclosed human-serum albumin NPs with the brand name Abraxane have reached an estimate of $967 million in revenue (Rossi and Rainer, 2020). After the first approval of Doxil^®^ (liposome loaded with doxorubicin with a size of ∼100 nm) in 1995 by the USFDA, research on nanomedicine grew exponentially across the scientific communities and current search on Clinicaltrials.gov resulted in 438 studies on various indications including oncology, autoimmune disorders, infectious diseases, cardiology, hormonal impairments, and orthopedics in different stages of clinical trials proving themselves to make a better world tomorrow (Clinicaltrials.gov). However, there is a need to shrink the gap from bench side to industrial production and comfortably reach the clinical applications (Boisseau and Loubaton, 2011; Khorasani et al., 2014; Hua et al., 2018). The literature for this review was largely gathered from different search engines like Google Scholar, Science Direct, PubMed, etc., with relevant search strings. We used the search terms “Nanotoxicology”, “Adverse outcome pathways”, “reproductive toxicity + nano”, “developmental toxicity + nano” “Microfluidics”, “Bioprinting”, “Stem cells in nanotoxicology”, “Advancements in nanotoxicology”, “organ-on-chips”, “Omics”, “Artificial intelligence and Machine Learning in nanotoxicology”, “Episkin models”, and relevant articles published in a time period between 1986 and 2020.

### Origin of Nanotoxicology

Nanoparticles, nanomaterials, nanosystems, nanoformulations, and nanomedicine, for example, are thought to operate differently from the bulk substance of the same chemical compound. Due to their tiny size and large surface area, these NPs interact with the biological system far more powerfully than bulk materials. Contrary to the beneficial biological effects most often they may result in causing adverse effects, study of these impacts is termed nanotoxicology. Therefore, it is necessary to look at the safety concerns of NPs, while establishing them for different applications. Nanotechnology and nanotoxicology are thus considered to be two sides of the same coin as the same nanosize which offers plenty of beneficial effects may also pose unwanted adverse effects. With the novel concepts of nanodrug approaches, tremendous applications and increased acceptance of these products were gained, while the safety of NMs is still a concern with a lack of sophisticated tools in evaluating their toxicity issues (Linkov et al., 2008). Some of the nanomedicine applications of the potentially active materials are hindered because of their ineffective target binding and other detrimental effects. If such detrimental effects are not identified or addressed properly, the development of the future generation of nanotechnology may be impeded and ultimately may pose risk to the development of science and technology (Viswanath and Kim, 2016; Mohanta and Ahmaruzzaman, 2020; Sahu and Casciano, 2009; Ren et al., 2016; Kaundal et al., 2017). From reported cases, it is observed that the lung and heart are the major organs that are often affected by NPs as these NMs mimic the air pollutants, easily airborne and distributed widely in the lung regions resulting in pulmonary and systemic effects. The effects start with inflammation and oxidative stress, are directed towards fibrosis, granuloma, coagulation issues, and cardiac disturbances, and ultimately lead to organ damage (Wani et al., 2011; Sarkar et al., 2014; Khanna et al., 2015). This is endorsed with the inhalational effects of multiwalled (MWCT) and single-walled carbon tube- (SWCT-) induced platelet aggregation effects in experimental animal models which were similar to humans (Du et al., 2015; Gu et al., 2015). Upon following the reports that cationic substances interfere with blood clotting, NPs originated from gold and polystyrene displayed similar toxic effects (Casals et al., 2012; Libralato et al., 2017). Therefore, it can be accepted that nanotoxicology is still a developing area due to the lack of standard protocols for assessing the toxicological concerns of NMs (Casals et al., 2012). Further, consistent reproducible methods for the safety evaluation of NPs must be developed (Mitjans et al., 2018). Once adequate protocols and assays are developed, suitable nanomaterial safety guidelines can be framed for the harmonization of risk assessment. Nevertheless, some authorities like the National Institute for Occupational Safety and Health (NIOSH), Industrial Technology Development Organization (NEDO), and American Conference of Governmental Industrial Hygienists (AGGIH) have reported few guidelines for NMs and provided occupational exposure limits (OELs) which can reduce the risk of toxicity (Ellenbecker et al., 2018; Rodríguez-Ibarra et al., 2020).

### Common Mechanisms Involved in Nanotoxicity and Assays Used to Evaluate Nanotoxicity

The future vision of nanotechnology in the medical field will get brighter with the improved and successful development of nanomedicine with minimal to mere toxicity concerns (Sahoo et al., 2007; Rodríguez-Ibarra et al., 2020). Thus, nanopathology resulting due to nanotoxicological effects can become a significant interest of research (Montanari and Gatti, 2016). It is necessary to figure out the various spectrum of toxic effects of any NMs or NPs that might produce upon intentional use or inadvertent exposure (Gatti and Montanari, 2018). To rule out the toxic effects of any NMs, it is essential to understand the simple to complicated mechanisms associated with nanotoxicity outcomes. Based on the previously published literature, it was generalized that inflammatory stimuli, inflammatory cytokines overproduction, increased reactive oxygen, and nitrogen species production (RONS) are observed with most of the NMs-induced initial toxic effects, en route to any of the apoptosis, necrosis, and autophagy-mediated cell death mechanisms, ultimately leading to cytotoxicity (Casals et al., 2012; Fu et al., 2014; Khanna et al., 2015). Further, the development of oxidative stress with antioxidants depletion (Akhtar et al., 2010) and interaction with oxygen-containing ligands considered (forming free radicals with stable S- and N-bonds) were found to be another mechanism for nanotoxicity (Madkour, 2020; Lippert et al., 2011). NPs differ from other biopharmaceuticals and small molecules in inducing toxicity despite the same size and chemical composition. This is because of their tiny size, the surface area increases exponentially, and thus reactivity increases causing band gap alterations to decrease melting point cumulatively causing serious side effects. Besides, differences in particles sizes also exhibit differences in mechanism to reach cells and distribute.

**FIGURE 1:**
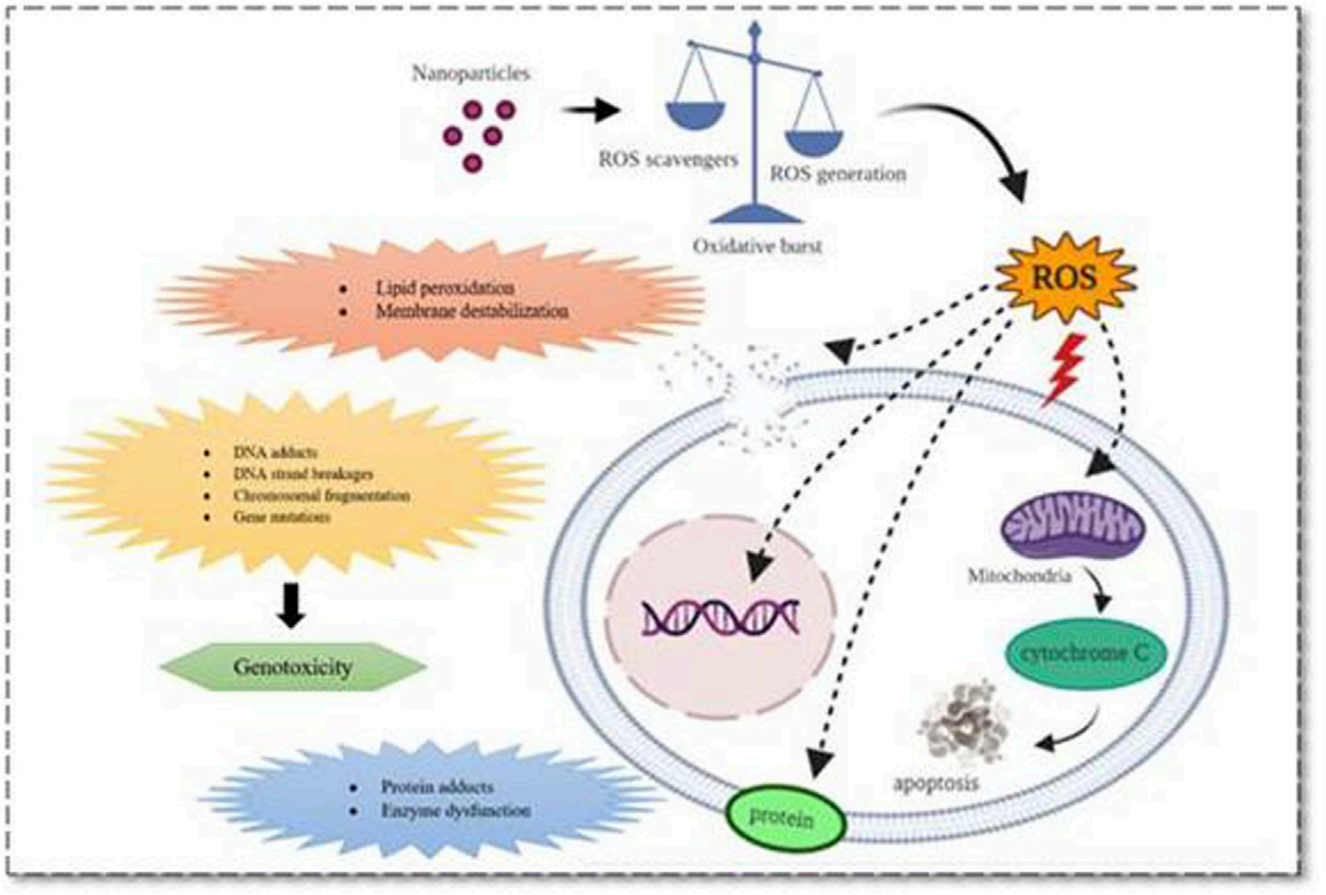
Various mechanistic pathways (molecular level) that can alter the physiological functioning of cells upon nanoparticle interaction which can either induce or release cell damage constituents. These mechanistic pathways that crosstalk with various organs and cells including immune cells and thus regulate pathogenesis, progression, and death of a cell were represented.

From the reported studies, the role of apoptosis in nanotoxicological effects with its common pathological role in mitochondrial dysfunction was majorly seen during redox species (ROS) generation. Also, dysfunction of mitochondria leads to endoplasmic reticulum (ER) stress, lysosomal dysfunction and therefore affecting the normal functioning of vital organs with the aggregation of unfolded proteins during cell rescue mechanisms (Ou et al., 2017; Ee et al., 2018; Rathore et al., 2019; Wang et al., 2015a; Li and Ju, 2018; Figure 1).

These events, in turn, initiate all three cell death mechanisms, i.e., apoptosis, necrosis, and autophagy. Besides lysosomal dysfunction, necrosis is also a common observation that occurs due to cytosolic acidification (Mohammadinejad et al., 2019). As mentioned earlier, inflammation has an equivalent role with oxidative stress in producing nanotoxicity with the involvement of immune regulatory molecules (Li et al., 2014). Practically this was proved by numerous NPs including carbon nanotubes and fullerene derivatives in various animal models (Fisher et al., 2012; Yanamala et al., 2013). So far the noted events are known to be involved and produced crosstalk in nanotoxicology effects is nuclear factor-kappa B (NF-κB), hypoxia-inducible factor (HIF-1), phosphoinositide 3-kinase (PI3-K), and mitogen-activated protein kinase (MAPK) pathways (Pacurari et al., 2008; Saifi et al., 2018a). Zinc, cadmium, silica, and iron NPs produced nanotoxicity effects with impaired NF-κB signaling, and SWCTs (0.8–2 nm) produced the toxic effects through PI3-K/Akt/mTOR pathway (Pacurari et al., 2008; Pinsino et al., 2015).

Further, NPs have been proven to cause cerebral toxicity depending upon their surface charge, thereby altering the integrity and distribution in the brain. However, these two hypothesized mechanisms provide NPs access to the brain despite the close connections and restricted entrance across the blood-brain barrier (BBB): 1) Transport through transynapse after inhalation, majorly observed with carbon, Au, and MnO2-based NPs (Raj and Kumar, 2020). This entry initiates ROS generation and pathogenesis of existing Parkinson's and Alzheimer's illnesses will be worsened later. 2) Another possibility is through uptake by BBB (Zhou et al., 2018). This was observed from the modified structure of NPs during drug delivery designing, i.e., the inclusion of either a high concentration of anionic or cationic NPs that may be toxic to the BBB. Based on the previous reports, we have listed the most common toxic effects observed with NMs employed in nanomedicine applications and illustrated in Table 1

**TABLE 1:** Nanotoxicological outcomes of some commonly employed NPs in nanomedicine

S. no.	NPs	Therapeutic applications	Reported toxicities	References
1	Fullerenes	• Antimicrobial agent	• Ecotoxic via effluents	Montellano et al. (2011); Djurasevic et al. (2019)
		• Carrier for gene and drug delivery system	• Impairing the redox balance in the brain and producing reactive fullerene metabolites by cytochrome P450 metabolism	
2	Carbon nanotubes	• Drug delivery	• SWCT: generation of reactive oxygen species, oxidative stress, lipid peroxidation, and dysfunction of mitochondria, along with cell morphological changes when incubated in epithelial cells	Manna et al. (2005); Luanpitpong et al. (2014); Dong (2020)
		• Biosensing	• MWCT: chronic inflammation of lungs and fibrosis and granuloma formation	
3	Quantum dots	• Medical imaging	ROS induction, impairing the functioning of mitochondria and nucleus by damaging the plasma membrane	Tang et al. (2008)
		• Diagnostic agent		
		• Drug delivery		
		• Gene therapy		
4	Gold NPs	• Drug delivery	Gold nanorods exhibited cytotoxic effects	Parab et al. (2009)
		• Theranostics		
		• Photothermal therapy		
5	Silica	• Drug delivery	Cytotoxic via an increase in ROS and a simultaneous decrease in glutathione levels	Wang et al. (2009); Liu et al. (2019)
		• Theranostics		

Also these reports now seriously warn us to concentrate on understanding the physical or chemical characteristics of the NMs that can help us to understand toxicities arising at bio-nano-interface which help in minimizing the nanotoxicity.

### Conventional Methods Employed for Nanotoxicity Evaluation

As part of routine toxicity evaluation of NPs, cell-based *in vitro* assays are employed to predict the toxicity before subjecting to animals, thus minimizing their utility (Casals et al., 2012). These assays provide advantages of animal-free procedures and inexpensive and direct methods with a simple endpoint in the form of colorimetric, fluorescent, and luminescent observations (Keene et al., 2014). However, interference of the chemical reagents used in these assays with NPs produces inappropriate results with misinterpretations (Hartung and Sabbioni, 2011; Greish et al., 2012). In Table 2, we have compiled the commonly employed conventional methods that are practiced for evaluation of nanotoxicity effects along with the concerns which warrant more advanced and specific techniques for assessing the toxicity of NPs.

**TABLE 2:** Table enlists the common disadvantages associated with the routine cytotoxicity evaluation methods

S. no.	Nanotoxicity evaluation	Mechanism	Methods	Concerns	References
1	Cytotoxicity	Metabolic activity	• MTT	Not sufficiently sensitive for detecting viable cell number and dye interference with NPs	Tournebize et al. (2013)
			• XTT		
			• Neutral red dye		
			• Resazurin		
			• NRU assay		
		Membrane integrity damage	• Trypan blue (TB)	Low sensitive technique cannot be used individually (in the case of PI and AO)	Aslantürk (2018)
			• Propidium iodide (PI)		
			• Adverse outcome (AO) staining assays		
		Apoptosis	• TUNEL annexin-V	False-positive results in identifying necrotic cells and cells which are undergoing DNA repair or gene transcription	Mccarthy and Evan (1997)
			• Caspase assays		
		Proliferation assay	• Thymidine (3H-TdR)	Need of radioactive compounds and also Requires harsh treatments of tissue sections	Tan (2019)
			• Bromodeoxyuridine (BrdU) assays		
2	Genotoxicity	DNA damage	• Single-cell electrophoresis	Lack of specific protocols and automated assay methods	Nandhakumar et al. (2011)
		Chromosomal damage	• Cytokinesis-block micronucleus (CBMN) and chromosomal aberration assays	Cannot differentiate between dividing and nondividing cells	Song et al. (2017)
3	Immunotoxicity		• ELISA	• Labor-intensive and expensive	Hosseini et al. (2018)
			• RT-PCR	• Insufficient level of sensitivity	
5	Oxidative stress	Depletion of antioxidant capacity	• GSH	• Indirect methods	Love et al. (2012)
			• DCFDA		
			• MitoSOX		
6	Inflammation	Inflammatory cytokines	• Release of inflammatory mediators like nitric oxide inflammatory cytokines	• Need of dedicated ELISA kits	Mitjans et al. (2018)

## Novel Methods Employed for Nanotoxicity Evaluation

Due to several problems associated with routinely used models and assays for NPs safety evaluation, the outcomes of NMs safety studies were quite inconsistent and results were highly varied from study to study and laboratory to laboratory. Further, it was also felt that there is a need to develop novel unconventional methods and assays for accurate and consistent evaluation of NMs safety. In the following sections, we have included some of the important and promising assays proposed for NPs safety evaluation.

### Cytotoxicity Evaluation

Even though there are many standard assays available and utilized for NMs cytotoxic effects, most of these assays require chemical reagents to evaluate the cellular metabolic conditions. Unfortunately, these assay reagents often interact with different NPs and can influence outcomes. Also, the interaction of cell culture media with NPs was reported with false-positive toxic effects. The following sections cover various novel assays employed or proposed for NMs safety and toxicity evaluations.

#### xCELLigence

An *in vitro*, noninvasive toxicity assay method provides an opportunity to observe all the events of the cell growth, i.e., real-time tissue cells, cell growth, cell proliferation kinetics, cell size, reproduction, and morphological effects with its label-free techniques which can avoid interaction of chemicals, dyes, and other cells as observed in other conventional cytotoxicity methods (Özdemir and Ark, 2013). This method can thus rule out false-positive and false-negative results as observed in other NPs toxicity assay methods. Also, this method is considered effective because of its electrical impedance tool which quantifies cell proliferation/viability, morphological changes, and attachment (Kustermann et al., 2013). Further, Scott Boitano Research Group at the University of Arizona studied the toxicity of 11 different inorganic NMs (AgO, Fe_2_O_3,_ Al_2_O_3_, ZnO, CeO_2_, FeO, Mn_2_O_3_, SiO_2_, TiO_2_, and ZrO_2_) and compared them with the conventional methods (MTT assay) in 16HBE14o cell line (Stefanowicz-Hajduk et al., 2016). On the experimental grounds of working, cells of interest are platted in an electronic microtiter plate (E-Plate^®^). Upon adhesion, cells impede the flow of electric current from electronic sensors produced located at the bottom of each well, and the impedance value is expressed in terms of a Cell Index (CI). The results obtained are directly proportional to the sensing electrode exposure with time reaching to plateau as the cells proliferate and reach 100% confluence. This assay can be considered as a sensitive and precise method to detect cytotoxic effects with continuous data acquisition for multiple studies (Ke et al., 2011). Therefore, these models can be effectively used for the accurate evaluation of NMs-induced toxicity effects and high throughput is also possible with these systems.

NMs undergo nanospecific interactions by acting as quenchers or enhancers besides absorbing or scattering light and thereby reacting with assay reagents, thus making toxicity determination even more challenging. The absorption and scattering that deform information flowing from the item are a key challenge in focusing on the internal architecture of tissues. In this regard, some technologies to decrease the dispersal effects via nonlinear light interaction, either using light microscopy by constraining the light exciting area to a selective layer or two-photon microscopy, have been developed. However, in the majority of applications, staining of samples also may not be achievable, Therefore, label-free methods have been designed depending upon optical properties, such as optical projection tomography and Raman scattering-based methods such as tip-enhanced Raman spectroscopy (TERS), surface-enhanced Raman spectroscopy (SERS), and shell-isolated nanoparticle-enhanced Raman spectroscopy (SHINERS). In surface-based vibrational spectroscopy, the Raman intensity increases by 10^14^ times when Raman molecules are near the noble metals like gold, silver, and copper with rough surfaces which possess a unique property called “localized surface plasmon resonance” (Israelsen et al., 2015). This phenomenon of enhancement of Raman scattering in the presence of gold and silver nanoparticles is termed “surface-enhanced Raman spectroscopy” (Israelsen et al., 2015; Zhang et al., 2019), and this effect is because of various factors like nanoparticle size, shape, surface properties, and configuration (Navas-Moreno et al., 2017). In a study reported, researchers evaluated cytotoxicity of TiO_2_ nanoparticles and single-walled carbon nanotubes (SWCNTs) on two types of cell lines, A549 (human Caucasian lung carcinoma) and HSF (human skin fibroblast), in which gold nanoparticles are used as SERS-substrates (Kuku et al., 2016). In case of TERS, generic substrate has a substance attached with the probe, where a nanoscaled gold tip present on the substrate functions as a Raman signal amplifier (Stöckle et al., 2000). But the signal generated from the gold tip is rather weak making it a major drawback of this technique. Another kind of substrate enhanced Raman spectroscopy is SHINERS. In this phenomenon, the intensity of Raman scattering is amplified by the plasmonic nanoparticles which acts as electromagnetic resonators that notedly increase the electromagnetic radiations from the electric field (Li et al., 2010). An ultrathin monolayer of such noble metal nanoparticles like SiO_2_, MnO_2_, etc., is dusted over the surface of the probe which slightly dampers the electromagnetic enhancement but also keeps the NPs away from forming agglomerates and prevents interacting with probe directly, because direct contact with probe may lead to change in structure of biomolecules (Fang et al., 2015). Very few researchers used these models and reported SERS as a fair alternative approach to probes with fluorescent property for biolabeling due to their photo stability and capability of multiplexing (Navas-Moreno et al., 2017). Although fluorescence-based screening techniques provide signal specificity and automatic evaluation of a large number of samples, they also have drawbacks, such as the need for exogenous labels, which may compromise cell integrity, the delivery of probes, the need for selective plates, and delayed focusing of image (Bortner and Cidlowski, 2004). Autofluorescence can also be utilized as a label-free fluorescent technique. Through an optical or electrical inducer, these biosensors transform the cell stimulation into a cell-created measurable signal. Some devices, like the Epic and EnSpire, employ resonance waveguide gratings to create an evanescent wave that detects entire cellular responses. To detect cell responses, other commercial devices such as ECIS, xCELLigence, and Cell Key depend on a low electrolyte impedance interface (Bortner and Cidlowski, 2004). However, none of the label-free methods have enough spatial resolution at the single-cell level. In this regard, scan-free technologies such as digital holographic microscopy have been intended to retrieve the wavefront object, resulting in a layered picture of an object through digital focusing and topographic image (Fang et al., 2006). In combination with optical sectioning techniques and digital holographic refocusing, the dark-field technique has been demonstrated to be promising in enhancing image contrast for interior layers. Dark-field digital holographic microscopy, a label-free technology most suited for image-based examinations, was developed to address this problem. The signal is generated using biophysical parameters such as absolute cell volume, transmembrane outflow, dry mass, protein concentration, and permeability (Mcguinness, 2007). Therefore, it is considered the finest noninvasive imaging tool for identifying numerous processes in a cell such as cell migration, differentiation, and death. This technique produces photos with an extended depth of focus (Kühn et al., 2013). Digital holographic microscopy has recently been used in live-cell imaging, early cell death detection, cell water permeability, and the analysis of toxin-mediated morphology of single cells (Neumann et al., 2006). For instance, loss of cell volume or cell shrinkage during apoptosis is a major distinguishing trait from necrosis, which is defined by initial cell swelling. However, the modest changes in cell volume can be regulated by their own regulatory mechanisms to maintain a balance of ions across the membrane. But in these cases, the inability of cell to regulate either by inactivation or overridden of regulatory mechanisms will subsequently activate cell death processes. Dark-field digital holographic microscopy is utilized in this case to track early cell volume regulation in response to events that are likely to cause cell death (Kühn et al., 2013). Dark-field digital holographic microscopy is another *in vitro* based technique for rapid assessment of cell viability by dynamically or quantitatively measuring shape and volume with high sensitivity.

### Measurement of ROS Levels

**FIGURE 2:**
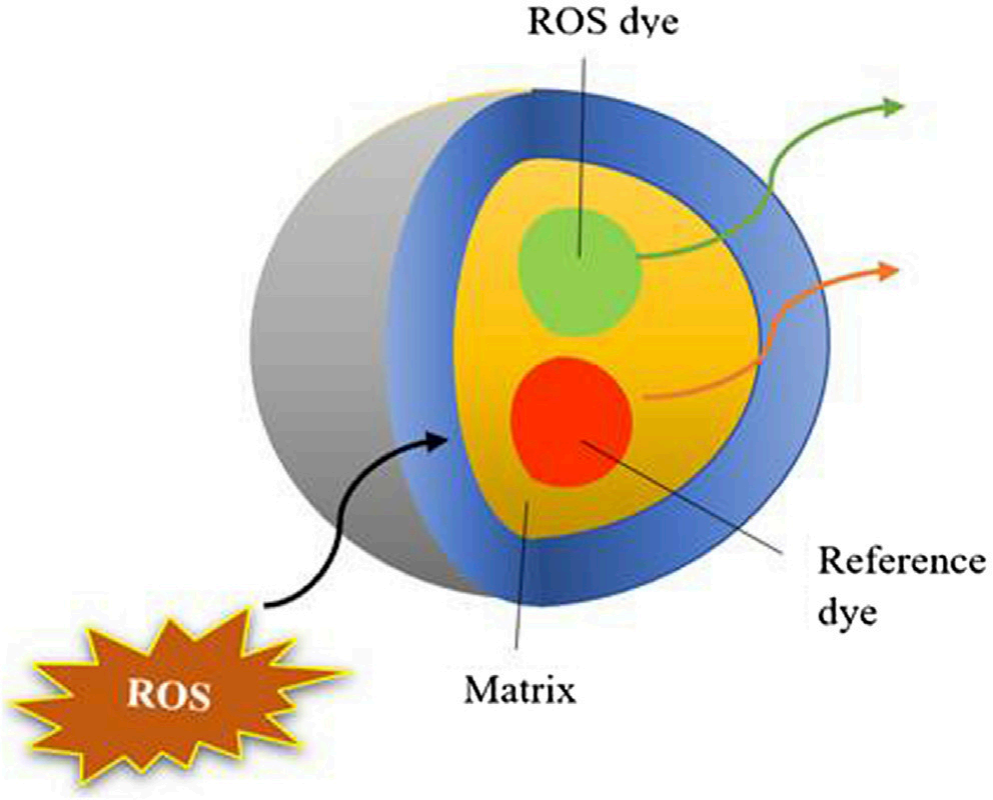
Pictorial representation of fluorescent dye utilized for assessing nanotoxicity.

During aerobic respiration of mitochondria, produced ROS initiates the mitochondrial damage and is a key regulator involved in a wide array of toxicological mechanisms, responsible for the pathogenesis of diseases. Due to this, monitoring and regulation of ROS levels have become an essential tool in research communities. As discussed in the above sections the prominent role of NMs in the production of ROS and its consequences, nanotoxicity assessment is therefore highly recommended (Figure 2). Despite these conventional techniques like LDH, MDA, dihydrorhodamine 124, DPPH, DCFDA, nitric oxide, etc., and some new techniques if any are highly appreciated to overcome present-day obstacles. Hence, to understand and measure these ROS dynamics, novel precise tools and assays are constantly being developed. Here, in the current review, we have discussed the new techniques that are recently developed for measuring ROS along with their advantages over conventional methods.

#### Fluorescent Probes for ROS Measurement

To overcome the concerns arising from ROS detecting fluorescent dyes, intensive research was carried out in developing advanced techniques, which witnessed advanced fluorescent probes like boronate-deprotection probes and NO-specific probes today. The ability of H2O2 to easily react with boronate groups is used in the development of boronate probes for the accurate exploration of ROS intracellular signaling (Woolley et al., 2013). These probes (peroxyflour-3; peroxy yellow) detect changes in H_2_O_2_ concentration upon epidermal growth factor (EGF) stimulation (Dickinson et al., 2010; Underwood, 2019). The acetoxymethyl compound enhances dye cellular retention and hence enhances efficiency to H2O2, in addition to offering a longer imaging facility (Lippert et al., 2011; Woolley et al., 2013). Although H_2_O_2_ localization studies and quantitative analysis of H_2_O_2_ are improved, there left scope for further development as these probes were single wavelength emitting (Woolley et al., 2013). To address this, a monoborate-based probe, Peroxyxanthone-1, is designed, which is the first-generation probe of this kind that depends on chemo selective boronate deprotection rather than nonspecific oxidation to provide an optical response (Miller et al., 2007). Later, Redoxfluor- (RF-) 1 was developed to detect various reversible redox processes in the cell (Miller et al., 2007). The incompatibility of these probes with animal models is their primary flaw; i.e., diffusion of probe, organ and tissue penetration, and subsequent imaging cannot be managed (Woolley et al., 2013). To avoid these issues, near-IR detection of the cyanine-7 with chemoselectivity of phenyl boric acid was designed. Due to NIR photons’ great penetration and minimal background fluorescence, it is gained as an efficient tool in *in vivo* investigations (Woolley et al., 2013). Similarly, peroxy caged luciferin was designed for noninvasive ROS detection in live mice (Bhatt et al., 2012; Woolley et al., 2013). Following, Mitochondrial Peroxy Yellow 1 (MitoPY1), SHP-Mito, and Mito-B have been generated for mitochondrial targeting (Woolley et al., 2013; Ribou, 2016). In recent years, fluorescent probes offered an excellent level of sensitivity and accuracy in measuring cellular redox dynamics. However, due to their irreversibly oxidizing mechanism, these probes are minimally used (Woolley et al., 2013). Due to these unique properties offered by these novel ROS detecting systems at *in vitro* and *in vivo* levels, the ubiquitous NP mechanism has had harmful consequences in the form of oxidative stress which can be better evaluated for a wide range of nanoformulations and NMs.

NPs are more likely to interfere with fluorescence testing due to their distinct physical and chemical characteristics and enhanced reactivity. NPs exhibit a wide range of optical characteristics that substantially differ from optical qualities displayed by identical bulk material. When light is incident on NPs, it can be either scattered or absorbed depending on their particle diameter. Extinction is due to absorption at diameters less than 20 nm, whereas extinction is caused mostly by scattering at sizes more than 100 nm.

#### Genetic Approaches for ROS Detection

Tackling the irreversibility concerns of fluorescent samples, more advances in detection methods were put in front with genetically encoded reporters, which can target specific cellular compartments. It was demonstrated that genetically modifying cells to create a redox-sensitive fluorescent protein may be used as an alternative to fluorescent dyes. The primary benefit of the genetic method is reversible oxidation, which allows for dynamic ROS monitoring. However, when compared to traditional fluorescent dyes, genetic alteration is not always feasible or simple (Woolley et al., 2013).

#### Nanoprobes for ROS Detection

Nanoprobes are designed by enveloping the dye in a nanoparticle delivery system that was designed to address the flaws of traditional fluorescent dyes (Woolley et al., 2013). Conventional dyes are subjected to nonspecific interaction following drug delivery into targeted cellular organelles and show potential cytotoxicity, which can be overcome by nanoprobes development. This is because probes are enveloped in a matrix of chemically neutral material (PVC, polyacrylamide, and gold colloid), which shields from nonspecific interactions and does not exhibit any cytotoxic effects. As their size is sufficiently small, they can be readily injected into cells using conventional methods such as microinjection, lipofection, and TAT-protein delivery (Woolley et al., 2013). The first nanoprobe was designed as Photonic Explorer for Biomedical use with Biologically Localized Embedding (PEBBLE) with 20–600 nm in diameter (Koo et al., 2007). Recent advancements in NMs have opened up a new path for the creation of optical biosensors based on carbon nanotubes, allowing for multimodal monitoring of a variety of ROS.

#### Nanoelectrodes for Measurement of ROS in Superparamagnetic Iron Oxide Nanoparticles

In recent years, magnetic NPs such as superparamagnetic iron oxide NPs (SPIONs) grabbed more attention in nanomedicine for their possible diagnostic and therapeutic applications. Till now, SPIONs such as magnetite, maghemite, and Fe_3_O_4_ are only magnetic NPs approved for clinical use. Optical methods are unable to detect ROS in a single cell and also cannot be measured over long periods due to the fast inactivation of fluorescent dyes (Erofeev et al., 2018). In this case, electrochemical sensor systems can be the best choice because of their portable size, cost-effectiveness, and feasibility in *in vitro* and *in vivo* assessment. Electrochemically reduced graphene oxide amperometric biosensor coupled with cytochrome C-modified glassy carbon electrodes has been developed to measure hydrogen peroxide and superoxide anions (Thirumalai et al., 2017). Due to its size and sensitivity, it is not suitable for single-cell analysis. Later, early nanopipettes were found to be the best alternative for measuring ROS within a single cell (Song et al., 2018). Actis et al. developed a disk-shaped carbon nanoelectrode with platinum placed on its surface; however, this was not successful due to the removal of platinum while penetrating the cell (Actis et al., 2014). To combat the drawbacks of previous nanoelectrodes, Erofeev et al. developed a probe and measured intracellular ROS by using novel carbon nanoelectrode with enhanced platinum adhesion based on quartz nanopipette. When HEK293 and LNCaP cells were exposed to 10 nm iron oxide NPs, the findings revealed a substantial variation in intracellular ROS levels (Erofeev et al., 2018). These tools have been proven to be an NP toxicity assessment technique in less than 30 min, as well as to be more sensitive and quicker than traditional commercial procedures (Erofeev et al., 2018).

### Genotoxicity Evaluation of Nanomaterials

It is a surprising fact that the same characteristics of the NMs that make interesting and advantageous in the medical field also create toxic effects. This is because NMs enter into cells, react with cellular components, and remain in cells leading to long-term toxicity. For an instance, NMs entered into the nucleus and interact with DNA, affecting its function by causing DNA breaks, altered bases, and chromosomal damage, and may also interfere with microtubules during mitosis causing clastogenic effects (Azqueta and Dusinska, 2015). Hence, genotoxicity measurement is crucial in assessing the safety of NMs. The first report of the genotoxicity of NMs came into light with the first report of fullerene in the year 2006. To assess the genotoxicity, a series of tests like AMES assay, COMET assay, chromosomal aberration assay, micronucleus assay, etc., are available. Despite this number of tests, none of them can completely be able to evaluate the genotoxic potential of NPs as they interfere with assay components. For instance, the AMES testing for genotoxicity of NPs is not recommended because of its limited penetration or no penetration through the bacterial cell wall. According to studies, several NMs have tested negative in the AMES assay and yet positive in *in vitro* mammalian cell testing (Doak et al., 2012). The interaction between cytochalasin B and NMs represents a stumbling block in the case of the *in vitro* micronucleus test (Doak et al., 2012). Cytochalasins B impede cytokinesis and create binucleated cells. Cytochalasins B also block filaments by which endocytosis is implicated (Pfuhler et al., 2013). In order to assure cell exposure to NMs in the absence of cytochalasin B, the modification of an *in vitro* micronucleus test is necessary. COMET assay, another majorly used *in vitro* method for genotoxicity evaluation of NMs, is hypothesized to interact with assay components. Some studies mentioned the presence of NMs in COMETs; it illustrates their existence during the experiment and suggests that they may have interacted with the bare DNA, causing artificial damage (Karlsson et al., 2015b). It is a surprising fact that there are no set guidelines that are available to perform these assays for NMs, while researchers perform these experiments based on modifying the first reported method. According to recent research, the inclusion of NMs in the gel has no effect on the COMET tail (Karlsson et al., 2015a). Recently, the efficiency of COMET assay was improved by the invention of COMET Chip, a 96-well microfabricated high-throughput platform by the Massachusetts Institute of Technology in Engelward Laboratory for evaluating nanomaterial induced DNA single-strand damage in single cells (Watson et al., 2014; Nelson et al., 2017). This system measures the DNA-protein cross-links, single-strand, and double-strand damage caused by nanomaterial exposures. It allows simultaneous assessment of different types and concentrations of NMs, thereby greatly reducing the workload, enhancing productivity, and reducing the experimental variabilities. Apart from this, DNA fragmentation assay and electron microscopy can also be used to assess several genotoxic platforms like COMET Chip assay, flow cytometry/micronucleus assay (Nelson et al., 2017), flow cytometry/H2AX assay (Nelson et al., 2017), Automated FADU (Fluorimetric Detection of Alkaline DNA Unwinding), Gene Chips (Wang et al., 2016), and G-banding analysis (Wang et al., 2016). The conventional FADU assay requires a large number of cells and manually operated systems which made it technically difficult to perform. Now, this conventional method is replaced by an automatic laboratory robot that provides flexibility with 100-fold reduced cell number, easy handling of samples devoid of agitation in a 96-well microtiter well plate (avoids the shear stress on DNA), accurate dispense of reagents, and temperature-regulated and full light protection every time (Moreno-Villanueva et al., 2011; Nelson et al., 2017). GreenScreen HC assay is one of the most widely verified assays for NM genotoxicity research. Another BlueScreen HC, a luciferase-based version of GADD45α reporter assay in a 384-well plate, was developed (Hughes et al., 2012; Simpson et al., 2013). GADD45α is a growth arresting, DNA damaging protein that gets activated upon different cell stresses (Nelson et al., 2017). ToxTracker reporter assay with high sensitivity and high-throughput screening is designed using the modifications of conventional genotoxic assays. ToxTracker test comprises a panel of six cell lines with embryonic mouse stem (mES) which contain various GFP tags for unique cell signals. The earlier version of the ToxTracker assay panel consists of two reporter cell lines (Nelson et al., 2017), while in recent studies, it has extended with six different reporter cell lines which can suspect ROS, unfolding of proteins, DNA damage, etc. (Hendriks et al., 2016). Another major advantage is that mES cells used in this assay are untransformed and show good sensitivity in detecting genotoxic and nongenotoxic substances. ToxTracker tests have been proven to be a fast, promising technique for evaluating the genotoxic potential of NMs.

### Immunotoxicity Evaluation of Nanoparticles

NMs do not even trigger inflammation since they evade the particle clearance processes like phagocytosis because of their nanosize (Dusinska et al., 2017). Self-proteins interact with NMs, causing autoimmune responses to the body (Dusinska et al., 2017). Immunotoxicity can be studied in *in vivo* models as they can fully study pharmacokinetics (ADME), the factors which play a vital role in showing immunological responses. However, when the 3R concept is taken into account, new *in vitro* techniques must be devised. *Drosophila melanogaster* has recently become quite prominent as a model for immune-nanotoxicity research (Ng et al., 2019). But there are certain limitations like body temperature, biochemical and genetic differences between humans and *Drosophila*, less complex adaptive immune system, cost-intensive, and maintenance of stock. Hence, while broadening the human relevance, the European Union Reference Laboratory for Alternatives to Animal Testing (EURL-ECVAM) proposed the usage of human cell lines (peripheral blood leukocytes, which may be easily obtained from donors, should be used as cell sources) as *in vitro* tests. In this model, high interindividual variability between blood donors and short primary cell culture survival time remained a concern. Recently, several researchers provided alternative approaches of validated cell lines like human Jurkat T-cell, human lymphoid T-cell (MOLT-4) or B-cell (IM-9), human acute myeloid leukemia HL-60 cells, and murine T-cells, along with sliced tissues to assess immunotoxicity of NMs (Sewald and Braun, 2013; Dusinska et al., 2017). Generally, cytokine expression is analyzed by using ELISA, flow cytometry, and RT-PCR. Because of these limited *in vitro* methods to predict immunotoxicity, complete toxicology cannot be studied (Drasler et al., 2017). However, no particular regulatory methodologies for measuring the immunotoxicity of NMs exist at this time. A battery of such novel and specific assays can predict the adverse effect that needs to be developed. Human-based skin explant assays have recently been created as a unique method for evaluating immunotoxicity (Ahmed et al., 2016; Ahmed et al., 2019) and they can be adapted to test NMs and nanomedicine (Dickinson et al., 2019).

ISO/TR 16197:2014 provides a description and collection of useful *in vitro* and *in vivo* toxicological techniques, including ecotoxicological nanomaterial screening. Toxicological screening assays provided in ISO/TR 16197:2014 can be used for early decision-making in research and product development, rapid input on potential toxicological/safety problems, and preliminary assessment of produced nanomaterials, among other things. This guideline is divided separately between screening methods related to humans and assays related to environment. ISO/TR 10993-22:2017 intended to describe the general framework and highlights marked that are important in biological assessment of medical devices consisting of or using nanomaterials, which can also be utilized to evaluate nanoobjects formed as a result of deterioration, wear, or mechanical treatment procedures (e.g., *in situ* grinding, polishing of medical equipment) on medical devices not made with nanomaterials. This document addresses the common pitfalls and hindrances while assessing nanomaterials when compared to bulk materials. No detailed testing protocols were included in this document. ISO/TR 21624:2020 provides a glance of many exposure systems and *in vitro* cell-based methods utilized in studies simulating the design of a toxicology inhalation investigation.

The ICH S8 guideline provides suggestions on nonclinical testing methodologies for identifying substances that may be immunotoxic, which will aid in immunotoxicity testing decision-making. This includes the standard toxicity assays which includes histology, hematology, clinical chemistry, gross pathology, organ weights as an initial step to consider the pharmaceutical product as immunotoxic, and supplemental immunotoxicity studies which include T-cell dependent antibody response (TDAR), immunophenotyping, NK cell activity assays, host resistance studies, macrophage/neutrophil function, and cell-mediated immunity assays to further confirm their immunotoxic potential. Recently, CFDA has released guidance for industry and other stakeholders on the safety assessment of NMs. This guiding paper aims to help industry and the other stakeholders to identify and build a methodology to evaluate the possible safety problems of NPs in cosmetic goods delayed hypersensitivity which is among the common problems in drug development pipeline leading to many withdrawals from clinical use. Historically, the guinea pig maximization test, Buehler’s test, local lymph node assay (LLNA), and local lymph node proliferation assay (LLNP) are used to predict delayed hypersensitivity. Recently, Dobrovolskaia et al. mentioned two more testing methods in their review, namely, human cell line activation test (hCLAT) and myeloid U937 skin sensitization test (MUSST or U-SENS), developed by European investigators to accurately predict delayed hypersensitivity of nanoparticles. In immunotoxicity research, *Dobrovolskaia, Moghimi,* and *Szebeni* did extensive investigations to develop the standardized methods and guidelines to use the immunotoxicity methods. They did an excellent work on investigating the effects of nanoparticles on the immune system, distribution, biocompatibility, immunological properties of engineered nanomaterials, and their mechanisms, including common pitfalls in nanotechnology, and addressed various challenges looking for novel solutions, standard guidelines for usage of various methods, and choice of selecting the best method to predict immunotoxicity.

### Carcinogenicity Evaluation of Nanoparticles

A major portion of nanomedicine is designed and developed for anticancer activity but the probability of causing cancer is also high with the NMs (Becker et al., 2011). The current epidemiological research on nanotherapeutic product carcinogenicity is inconclusive. The database needed to assess the carcinogenic risk of NMs is likewise insufficient. The assessment of carcinogenicity and its relevance to humans always remains uncertain with their qualitative and quantitative effects. In terms of qualitative terms, small size, absorption, retention duration, distribution after overcoming all biological barriers, and subcellular and molecular interactions all play a big influence. In comparison to the respective bulk material, the carcinogenic potential of the nanomaterial is considered to be greater because of its tiny surface area and its size; i.e., the carcinogenicity of nanomaterial and non-nano-scale (bulk material) is fundamentally different. As global production of NPs is progressing day by day, new NMs with improved properties are expected in the coming years. Hence, the susceptible NMs inducing carcinogenicity should be identified and exposure should be minimized. To minimize the exposure, there is no doubt that an immense necessity for investigations in the area of developing and standardizing testing methods is recommended. In recent years, an advanced technique, namely, cell transformation assay, had been used to detect the carcinogenic risk of NMs. This is a novel approach that can measure the ability of the cell to cancer cells in a single step despite its multistep conversion process. Briefly, in this procedure, cells like primary Syrian hamster embryo (SHE) cells or stable cell lines like mouse BALB/c-3T3 or C3H/10T1/2 are employed for their ability to transform into the phenotype of mammalian cells upon exposure to NMs. This also facilitates identifying the genotoxic carcinogens apart from nongenotoxic NMs (Steinberg, 2016). Endpoints for the safe NPs include unchanged morphology, with retained density-dependent growth and colonies formation, devoid of any crisscrossed cell or Piled-up cell foci, etc., (Sasaki et al., 2014). In 2015, the European Union Reference Laboratory for Animal Test alternatives also published a paper to test chemicals for carcinogenicity using an *In Vitro* Syrian Hamster Embryocell Transformation Assay (Drasler et al., 2017; Dickinson et al., 2019). Wunhak Choo et al. assessed the *in vitro* carcinogenic potential of Ag NPs by Balb/c3T3 A31-1-1 mouse model (Choo et al., 2017). Sighinolfi et al. evaluated the carcinogenic potential of metal NPs by using the BALB/3T3 cell transformation assay (Sighinolfi et al., 2016). Too far, only a few studies to assess the safety of NMs are available; however, future investigations are needed before issuing the final recommendation. In recent years, transgenic models are also widely used to predict carcinogenicity as they are useful for the study and prediction of the human response to chemical exposure (Gulezian et al., 2000). Tg.AC and rasH2 transgenic mice and p53+/− and XPA−/− knockout mice have been proposed in testing carcinogenic potential, though few studies are done to assess the NMs (Gulezian et al., 2000). Takanashi et al. and Ying Liu et al. evaluated the carcinogenicity of carbon nanotubes and Ag NPs by using transgenic model rasH2 mice, respectively, in their studies (Takanashi et al., 2012; Liu et al., 2020). However, the usage of transgenic models in predicting nanotoxicity is still in the budding stage and yet to be developed in recent years.

## Advancements in the Evaluation of Organ Toxicity by Nanoparticles

### Hepatotoxicity

**FIGURE 3:**
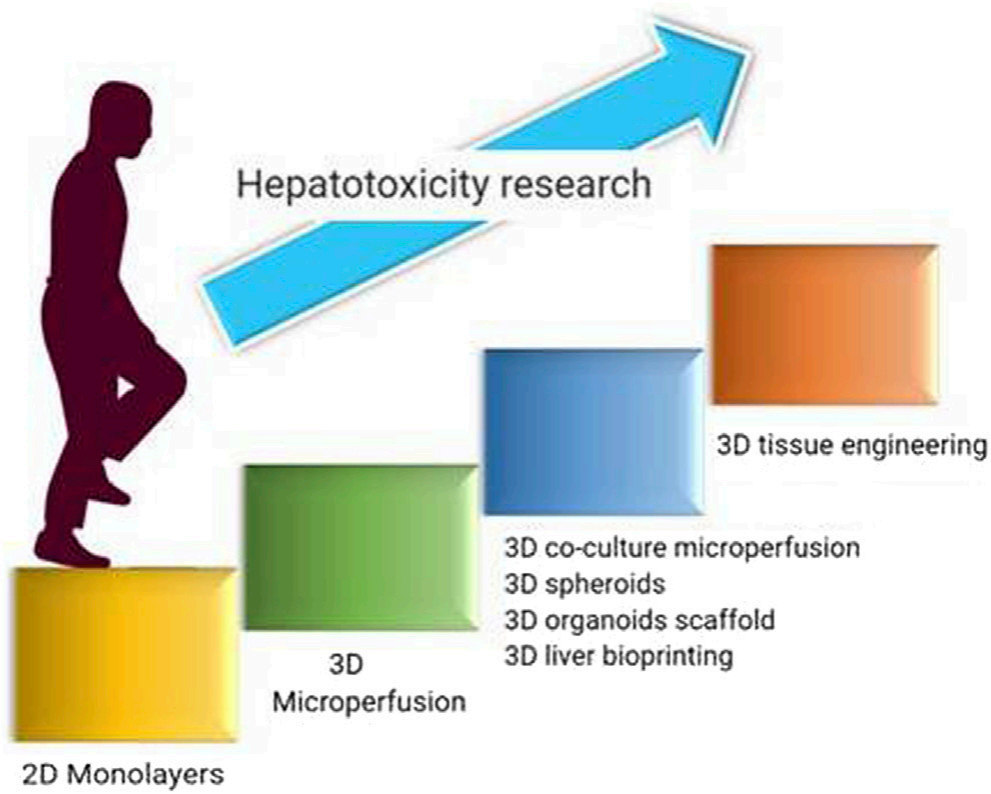
Developmental journey in advancements of hepatotoxicity evaluation employed for nanotoxicity evaluation.

Hepatotoxicity is the major concern with most of the drugs, and so with NPs even. This highly recommends evaluating the health status of the liver upon NPs subjection to humans. Conventional animal models are not suitable to accurately evaluate the hepatotoxicity as i) the data obtained by the *in vivo* studies cannot be extrapolated to the humans with certainty and ii) the hepatotoxicity observed in animal models is indirect and may be influenced by toxin bioactivation (Liu et al., 2019). The main effects of a drug-induced secondary effects compound in animal models are difficult to anticipate because of many endogenous and exogenic variables in liver function which lead to complicated interactions with organs (Liu et al., 2019). As a result, *in vitro* models offer a superior way to predict hepatotoxicity based on these parameters. Primary hepatocytes and hepatocyte-like cells (hepatoma cell lines, induced pluripotent stem cells, or stem cell-derived human liver cells) have been using extensively in the current research. Hepatoma cell lines like HepaRG and HepaG2 were isolated and grown from individuals with the disease. Apart from these cells, stem cell-derived hepatocytes such as embryonic stem cells (ESCs), pluripotent stem cells (iPSCs), human fetal hepatic progenitor cells (hFHPCs), and human skin-derived precursors (hSKPs) are also emerging as a potential source, as these cells closely resemble adult hepatocytes and are suitable for toxicity studies (Liu et al., 2019). They resemble hepatocytes with some limitations like loss of CYP450 expression, short-term utility, interdonor differences in primary hepatocytes, reprogramming changes induced during passages of iPSCs, limited genotypic variations, and ethical concerns made to identify and develop alternatives to predict hepatotoxicity (Deng et al., 2019). Few of them are in great progress in this field including 3D-bioprinting, organs on a chip, and organoids which are discussed in the following sections (Figure 3).

#### 3D Microfluidics

3D microfluidics is a technique for growing live cells or organs on a chip using microscale fluid manipulation. 3D microfluidics is a modified kind of photolithographic etching used to build microchips that provides the same surface characteristics and dimensions on the very same scale (nm to m) that live cells detect in their native tissue milieu (Bhatia and Ingber, 2014). It can miniaturize the cells or organs by a few square centimeters. Chambers are constructed by applying liquid polymers like poly-dimethyl siloxane on the silicon chip and polymerizing them in transparent rubber-like stamps to make them more biocompatible and flexible. The constant medium flow through the carriage of nutrients, metabolites, and oxygen provides a condition necessary to maintain the liver physiologically and functionally. Hepatocytes cultured using the microfluidics model showed good viability and proliferation. The design of a two-layer microfluidic device includes a parenchymal network in one layer and channels representing blood vessels in another layer. A nanoporous polycarbonate membrane separated these two layers, through which metabolites are transported and which also proliferates and functions for 14 days (Carraro et al., 2008). Lei et al. constructed a microfluidic 3D hepatocyte chip, which they described as a reliable and sensitive tool for studying NP hepatotoxicity profiles (Li et al., 2019). The human liver-on-chip is regarded as the greatest fit for testing on humans and the preclinical development stage of drugs. The microenvironment can be imitated by a single-cell culture but normally it is not enough to generate organ-like functioning. Therefore, the multiorgan microfluidic model that combines two or more different tissues with a dynamic flow of microfluidical connection between each separate compartment is being developed. The development of a multiorgan chip opens a great platform to perform *in vitro* repeat dose toxicity studies. Advancements in microengineering enable human-on-a-chip development, highlighting the relevance of many organ interactions in drug toxicity (Starokozhko and Groothuis, 2017). A four-organ chip was constructed with dynamically linked intestines, liver, skin, and kidney; however, no toxicity tests were carried out with this model (Maschmeyer et al., 2015). A novel method has been created which combines spheroids from chip and 3D culture with a continuous medium supply to the cells by osmotic pumping (Liu et al., 2019). In addition, cocultured spheroids may be used with multiorgan chips like neurospheres. As liver-on-chip technology is still in its emerging state, a number of nanotoxicity studies to support this concept have not been out. Their research application will undoubtedly lead to reduced animal usage, overall cost, and translation time to good preclinical predictions.

#### 3D Liver Bioprinting

3D printing of the liver is primarily related to digital model data. The objects are constructed by means of layer-by-layer printing of sticky materials such as digital light or powdered metal. The physical object is created from blueprint by superimposing the printed material layer by layer under electronic controls once the printer is linked to the computer. The structures of 3D printing are designed using the liquid inkjet binder onto the powder bed; hepatocytes and the culture medium are filled inside the 3D structures. This technique increases the liver-specific gene expression and CYP450 induction and improves morphological organization. 3D bioprinting has the advantages of precise control and customized design. The cells within a bioprint develop strong bonds with the extracellular matrix of each other and create soft solid microtissues nearly related to the natural liver. With the mentioned evidence, it can be suspected that 3D printing has great potential to study *in vitro* hepatotoxicity research and these systems can be explored for the evaluation of hepatotoxic effects NPs (Bogue, 2013; Liu et al., 2019).

#### 3D Organoid Scaffolds

The model of the scaffold is like the culture of isolated cells using a medium like Matrigel so that cells can grow in a three-dimensional manner (Liu et al., 2019). This culture system resembles *in vivo* tissues with the complex spatial shape of tissues and shows cell-cell and cell-matrix connections. When liver cells and nonparenchymal cells are seeded in the 3D organoid scaffold, they get attached to and start proliferating, eventually forming a functioning tissue (Ma et al., 2018a). These complex tissues were cultivated in multiwell plates or in circulating systems to assess the toxicity of new medicines (Liu et al., 2019).

In comparison with previous *in vitro* models, 3D bioprinting tends to provide numerous benefits. 3D bioprinting provides multicell directional control and displays a controlled deposition of various cell densities, making it the perfect method for architecting *in vitro* organ models (Gu et al., 2020). Microenvironment *in vivo* is far more intricate than 2D, in which 2D *in vitro* models show contrary results. 2D cellular models possess a lot of flaws which created a need to develop new 3D models; 3D bioprinting is good at it. Biosensors encapsulated in 3D microenvironments have the ability to monitor physiological processes in real time, toxins detection, and sophisticated diagnostics (Dias et al., 2014). Different bioprinting methods are constructed to address the challenges of different applications that possess their respective advantages. Nowadays, extrusion-based bioprinting is the most popular method of bioprinting. Industrial-grade extrusion-based bioprinters are usually more expensive, but they have greater resolution, speed, spatial controllability, and material versatility, albeit their precision is restricted to 100 nm (Gu et al., 2020). Inkjet bioprinting is the most cost-effective and accessible bioprinting method, with excellent precision, speed, and compatibility. However, it is difficult to print high viscosity materials or cells with high concentration, which reduces the structural strength leading to unsatisfied *in vitro* models (Murphy and Atala, 2014). 3D Bioprinting has low precision compared to natural organs due to the complexity of organs and tissues which makes accurate bioprinting greatly difficult. 3D printing has gone far not only in nanotoxicology but also in its applications. It is using extensively in many fields for assessing the toxicity of several drugs. It can also be used for organ transplantations which can contribute to huge shortage of organs for transplantation, but it is too optimistic due to complexity of human organs and unrevealed mechanism of organ growth (Murphy and Atala, 2014).

### Local Toxicity Assessment of Nanotoxicology by Episkin or Skin Ethnic Models

NPs formulations such as Ag NPs are being extensively used in the market nowadays because of their broad-spectrum antibacterial properties. Hence, the toxicity produced by using these products should also be of concern. The toxicity of Ag microparticles has been widely investigated in the last few years by using 2D-cellular models and *in vivo* models. Assessing the toxicity by using conventional *in vitro* and animal studies is producing conflicting results. This is due to the drawbacks of 2D dimensional cell cultures and an idea to replace animal studies by following the 3R concept. But 2D cell cultures lack the connections between cells and cell matrix, as seen in *in vivo*. There are no barrier functions in 2D cell cultures. As a result, 2D-cell cultures fall short of replicating the *in vivo* correlation. The use of animals might be limited by expense, biological safety, and animal problems in the field of toxicology (Chen et al., 2019). As a result, new *in vitro* models that accurately predict toxicity are in great demand in order to close the gap between *in vitro* and *in vivo* findings. Numerous techniques are under the developmental stage to create an environment that is similar to the native situations in *in vivo*. In that case, the present investigations focus on shifting from 2D to 3D in which there is an existence of extracellular barriers and cell-cell interactions that can mimic the absorption and distribution of materials. Such promising models include 3D spheroid culture systems, EpiDerm, and Episkin. 3D culture involves the embedding of cells in a gelatinous matrix to simulate the conditions where cells interact with the extracellular matrix (Lee et al., 2009). Because toxicity can be affected by the cellular environment, *in vitro* investigations of the biological effects of NPs using 3D model systems may be more suitable than using 2D appropriate models (Mueller et al., 2014). Lee et al. evaluated the toxicity of Au NPs in 2009 for the first time by using 3D cell spheroid models, and they noticed a substantial reduction in the harmful effects of Au NPs on 3D compared to 2D cells (Lee et al., 2009). As a toxicity assessment for the human epidermis, Liang Chen et al. developed a 3D epidermal model termed EpiKutis consisting of human keratinocytes. They concluded that the EpiKutis model, rather than 2D monolayers, was more likely to replicate genuine physiological reactions to AgNPs (Chen et al., 2019). Wills JW et al. assessed the genotoxicity of engineered NPs using a 3D *in vitro* skin model (EpiDerm) (Wills et al., 2016). This result shows that 3D epidermal models may be more suited to the assessment of skin-related NM risk.

### Phototoxicity Evaluation of Nanomaterials

Today nanomedicine is also developed to treat skin pathologies majorly as a carrier for natural medicines. During treatment with nanomedicine for skin disorders, there is a high chance of getting exposed to solar irradiation that may result in phototoxicity (Kim et al., 2015). Here, phototoxicity can be defined as light induced responses of the skin to photo-reactive chemicals (Choi et al., 2011). The mechanism behind this is the molecule of chromophore or photosensitizer when absorbing the photons produce a phototoxic reaction (Kim et al., 2015). Various test models have been established to identify the phototoxic potential of chemicals but mainly focusing on animal test methods; i.e., *in vitro* and chemico assays are widely used. Erythrocyte photo hemolysis, 3T3 neutral red uptake assay, and phototoxicity testing by availing human 3-dimensional (3D) epidermis models are the most used *in vitro* assays. Previoulsy, chemico methods that were employed for ROS and phototoxic risk assesments are same used for NMs phototoxicity assesment (Kim et al., 2015). This assay uses plasmid, but not live cells or tissues. It is another way to evaluate DNA strand-breaking activity by UV-induced phototoxic chemicals. However, these in chemico methods have limitations that include inapplicability for water-insoluble materials and lack of metabolic activation capacity. These models are only for risk identification, but not for the evaluation of phototoxicity potential (Kim et al., 2015).

Several *in vitro* tests have been rejected for use with drugs due to their hindrance at the clinical translation (ICH, 2015; Kim et al., 2015). Erythrocyte hemolysis is an *in vitro* test that uses the cell membrane of sheep red blood cells for the evaluation of photochemically generated ROS and radicals which cause hemolysis. This test has shown low sensitivity and its performance is not much superior compared to 3T3 NRU-PT (Kim et al., 2015). 3T3 neutral red uptake phototoxicity test (3T3 NRU-PT) is a widely used assay for soluble substances especially. 3T3 NRU-PT assesses photocytotoxicity by evaluating the cell viability in respective to chemical exposure upon the influence of light in the BALB/c 3T3 cell line (OECD, 2019). Though 3T3 NRU-PT has a high sensitivity, and if a compound exhibits positive results of phototoxicity, it should not be considered as an endpoint but should be recommended for further follow-upconformational studies.

To evaluate water-insoluble materials, novel rebuilt human skin models with a stratum corneum layer permitted the testing of various topically applied compounds. To assess phototoxicity, researchers employed assays built using reconstructed human skin to assess cell viability with and without radiation. Some tests, however, may be less sensitive than human skin *in vivo*, while the lowest positive reaction dosage might be very hazardous to human skin *in vivo*. Therefore, it is important to comprehend any selected assay sensitivity and its feasibility to adjust the conditions of assay accordingly. However, the lack of defined *in vitro* models for assessing the ocular phototoxicity is unexpected. Negative outcomes in the reconstructed human skin test and the 3T3 NRU-PT may indicate minimal risk of ocular phototoxicity (ICH, 2015; Kim et al., 2015).

## Novel Technologies Employed for Toxicity Evaluation

### Computational Models

The evolution of AI and ML gifted the computational tools to empower nanomedicine with a low cost and effective approach in testing the safety concerns. This safety profiling at the initial steps of drug discovery with the integration of information at various levels provides reliable outcomes and negatively impacts the failure of the drug in the drug discovery process. Understanding the science, limitations and opportunities behind this application is essential for utilizing it in maximum ways. Also, computational methods not only use the ligand-receptor docking concept but also consider the pharmacokinetic properties for exhibiting the results. Herein, we discussed the recent computational models that are applied for evaluating the nanotoxicity of NPs.

#### Quantitative Structure-Activity Relationship (QSAR) Modeling

Computational tools such as QSAR and nano-QSAR models (at nanoscale) reduce the time, cost, and resources that are consumed at routine nanotoxicity studies. These models are mainly used to establish a correlation between pharmacokinetic and pharmacodynamic data to *in vivo* application scenarios. Traditionally, biology-based mathematical models like the Bayesian model, Monte Carlo simulation, QSAR, and nano-QSAR are widely studied approaches for the assessment of nanotoxicology. For the past few years, QSAR was considered the most promising tool to predict toxicity. It was first developed in the 1960s for the safety assessment of pesticides. Later, due to the growth of the toxicology field, regulatory agencies like REACH encouraged the use of QSAR as a substitute for animal models. QSAR approaches predict the biological activity of a compound based on its physicochemical properties (surface charge, solubility, and aggregation) and molecular descriptors. A molecular descriptor can be considered as a number that describes a specific property which may be an experimentally determined or a calculated one (Buglak et al., 2019). The traditional QSAR model known as Hansch analysis works by assuming that biological activity depends on geometrical and physicochemical descriptors. Later, another approach called 3D-QSAR was developed by Cramer and coauthors in 1988 (Cramer et al., 1988) in which the spatial structure of molecules, interactions, and activity are considered. Although both models are based on large data sets, they failed to express the specificity of NPs as their exact structure is unknown. As a result of this occurrence, a new model known as nano-QSAR modeling was created. Nano-QSAR is a most universal model as it covers one-dimensional (1D), two-dimensional (2D), and three-dimensional (3D) approaches. It covers not only a receptor-based response but also cell-based and organism-based responses (Buglak et al., 2019). Among all the QSAR models, 3D nano-QSAR model was considered the best model to predict nanotoxicity. Nano-QSAR cytotoxicity models work on dual descriptors: enthalpy (related to bandgap energy) and electronegativity (related to stability). According to Frontier's molecular orbital theory, a larger gap between the lowest unoccupied orbital energy (LUMO) and the highest occupied orbital (HUMO) energy is shown to be less stable; this allows a high conductivity which leads to increased NM reactivity (Singh et al., 2020). The low-energy conformations docked into the ADME model were used to build 3D nano-QSAR. In a crystal, if the atoms are close to each other, it enhances the chances of overlapping the orbital energies and subsequently splits. The valence band and the conduction band are separated by an energy gap as a result of this. Overlapping of conduction bands indicates the cytotoxicity or other disruptive effects of NMs. But this model showed limited success for crystals. In order to assess the predictive ability of developed nano-QSAR models, researchers conducted a study in which different types of NMs (metal oxides, carbon nanotubes, fullerene derivatives, etc.) are tested and outcomes were compared with *in vitro* cell-based assays. The proposed model has shown regression correlation *R* ^2^ of more than 0.851 between experimental and nano-QSAR-developed models (Singh and Gupta, 2014). Later, the quasi-SMILES-based QSAR model was developed using CORAL software and the cytotoxicity was assessed. Nowadays, quasi-SMILES seems to be the most effective technique to assess toxicity, as it considers experimental conditions and empirical properties.

### Omics Technologies

**FIGURE 4:**
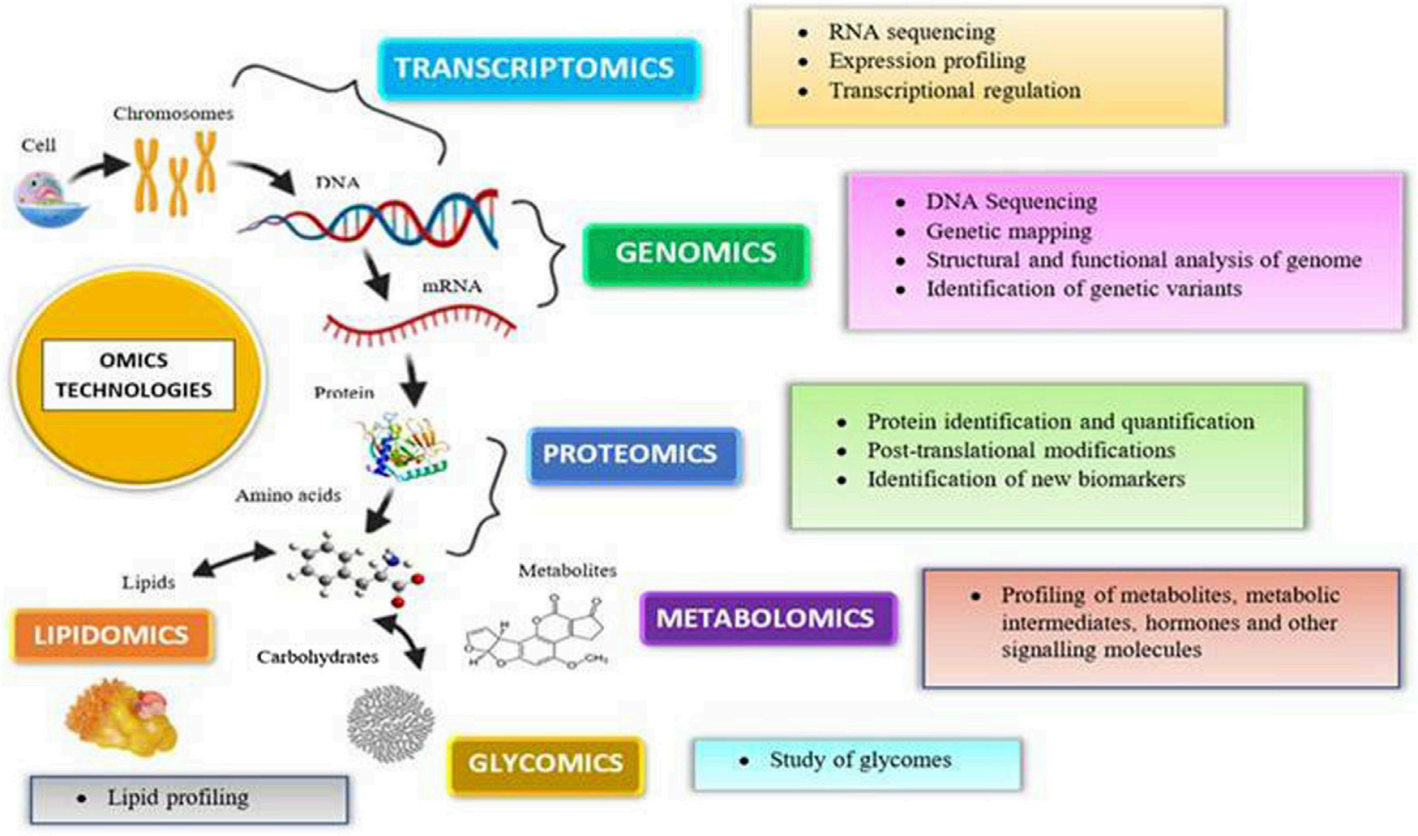
Various omics approaches utilized today for an assessment of nanotoxicity evaluations.

In *in vitro* and *in vitro* testing of NPs, core system technologies were also employed, namely, “omic” approaches (genomics, transcriptomics, proteomics, metabolomics, lipidomics, and toxicogenomics). The major advantages of such kind of technologies are that they may predict toxicity at low levels of nanoparticle exposure, which do not produce toxicity but can stress the cells; NPs cause less interference (no fake positive and negative results) observed with conventional methods. Even though *in vitro* models are highly regulated, they show false-positive results due to the interference of color, fluorescence, chemical activity, etc. (Froehlich, 2017). For example, NPs suppress the LDH enzyme’s activity, resulting in lower cytotoxicity levels than the genuine ones. This interference property of NPs is not confined to *in vitro* interference; it also interferes *in vivo*. Hence, the developed omics techniques gained popularity for their use in toxicity. Omics technologies require expensive infrastructure and highly skilled personnel to prepare the samples and to analyze the data. Proteomics helps identify new targets and biomarkers for nanoparticle toxicity. It not only provides information regarding the protein expression but also aids in the assessment of protein posttranslational changes. Proteomics has both technical and biological drawbacks, preparation can be contaminated, and protein expression changes with age, sex, and circadian rhythms (Froehlich, 2017). Genomics reveals the information regarding the epigenome that was altered by the toxicants, thereby helping in toxicity screening. Rather than screening chemicals for toxicity, it determines an individual’s susceptibility to a certain toxin. Metabolomics analyzes the endogenous metabolites present in the body after insult with a toxicant (Saifi et al., 2018b). Omics platforms could be useful in understanding the new pathways of nanomaterial toxicity, which is not possible in conventional methods. By providing precise and trustworthy data in a high-throughput way, omics-based toxicology screening will take toxicological research to a new level (Figure 4).

### Green Algorithms

The involvement of machine learning and artificial intelligence helps to accomplish more complicated and time taking tasks in less time. The discovery of an algorithm named “Hartung” brought new methodologies in the field of toxicology and was hailed as the software that can replace *in vivo* testing methods by supporting 3R principles. The algorithms work by generating a chemical map that contains hundreds of chemicals from the highest-predictability databases. The algorithm predicts toxicity by comparing and substituting chemical moieties inside the map with data from thousands of nanochemistry databases (Hartung, 2010). But currently, the emphasis on sustainable nanomedicine expansion is changing towards “green nanotoxicology” based on progress with ML toolbox and AI software more exact compared to animal tests in forecasting nanotoxicity” (Crawford et al., 2017).

### Stem Cells as a Novel Approach to Predict Toxicity

Pluripotent stem cells have the ability to develop into any type of cell in the body. Self-renewal and differentiation properties distinguish these cells, making them more distinct and potentially useful in regenerative medicine, developmental biology, and toxicity. Till now, oversimplified methods like 2D cell lines that lack accuracy are using in the testing and validation of compounds, so which majority of the exact NMs toxicity is still unpredictable. In the realm of toxicity, ESCs and iPSCs have received greater attention in recent stem cell research (Handral et al., 2016). ESC is produced from an embryo’s undifferentiated inner mass cells, which may grow into any tissue except nonembryonic tissues such as the placenta and umbilical cord. iPSCs are derived from somatic cells that have been reprogrammed to act as ESCs by turning on genes or forcing the expression of reprogramming genes such as Oct4, Klf4, and Sox2 (Takahashi and Yamanaka, 2006). For the first time in 1981, ESCs were extracted from mice (Evans and Kaufman, 1981). In the early 1990s, investigators started research by using mouse ESCs as an *in vitro* approach and reported the usage of stem cells in investigations of toxicology (Heuer et al., 1993). After 2 decades, in 1998, hESCs were isolated from the inner mass cells of the human embryo (Thomson et al., 1998). After that research on stem cells was extensively grown up in the field of regenerative medicine and still lies as the budding stage in the development of toxicological studies. Later, ECVAM (European Centre for the Validation of Alternative Methods) released funds to unfold an alternative platform made to set goals on the usage of hESCs in the era of toxicology. An embryotoxicity stem cell test (ETST) was designed and validated by ECVAM and successfully predicted the embryotoxicity by comparing hESCs results with *in vivo* models and characterized the chemicals based on their predicted toxicological effect. The results were reliable and it has been considered as a standard method to screen the embryotoxicity (Genschow et al., 2004). ESC-based Novel Alternative Testing Strategies (ESNATS) also commenced a cascade of protocols and assays to screen the different types of toxins (embryotoxins, cardiotoxins, etc.) (Kuske et al., 2012). First ever, a comparative study to evaluate the cytotoxicity of silver NPs was conducted by comparing the hESCs-derived fibroblasts with L929 cell lines and reported hESCs as the promising platform for future nanotoxicity screening. The cytotoxic potential of Ag NPs was verified in this study, which investigated nanoparticle uptake, apoptosis, cell differentiation, and cell cycle (Peng et al., 2012). Similarly, various sizes (1.5, 4, and 14 nm) of gold NPs were explored, and specifically, the core size of 1.5 nm was reported as highly toxic to cells. During the neuronal differentiation of hESCs, gold NPs caused epigenetic effects, and different sizes of NPs impacted DNA methylation and hydroxylation too (Senut et al., 2016). Unlike ESCs, the use of induced iPSCs is still in the infant stage. The utility of stem cells in the field of nanotoxicology still needs to grow extensively for enhanced toxicity evaluation.

### Electrochemical Approaches for Nanotoxicity Assessment

Conventional analytical techniques like microplate reader, cytometer, high content imaging, and spectrophotometric techniques typically usually take a long period of time and often lead to false-positive outcomes. Among various analytical techniques, bioelectrochemical techniques are able to measure the nanotoxic effects (*in vitro* and *in vivo*) by a noninvasive method, at multicellular as well as unicellular levels (Shah et al., 2014; Shinde et al., 2020).

Due to handling tiny sample volumes, simple instruments, ease of use, and point of care practicality, electrochemical analytical devices are extensively used. Nevertheless, this approach utilization in the assessment of intercellular, cell-drug interactions, and cytotoxicity is still at the infant stage. Electrochemical techniques give a boost in testing biochemical processes in cells and thus facilitating the information on kinetic parameters along with thermodynamics of cells under various conditions. Three important forms of electrochemical analysis are commonly employed in biological research in probing various cellular cytotoxicity events, for example, amperometric, potentiometric, and impedimetric testing.

Generally, viability methodologies depend on the activity of enzymes like proteases, esterases, and oxidoreductases and typically use optical methods like fluorescence, absorbance, and luminescence. Analytical chemistry is becoming a major tool in order to evaluate neurological and biophysiological changes in cells after contact with NM. Electrochemical molecules are analyzed easily by these methods and nonelectrochemical molecules analysis can be done by designing suitable biorecognition probes. This is mainly based on the concept of enzymatic changes in cells upon exposure to NMs.

Today electrochemical methods are widely used for studying the cell toxicity effects by NMs which involve various mechanisms like cellular exocytosis, RONS production, releasing of ions monitorization, and measurement of impedance behavior in cells, tissues, embryos, and whole organisms. Amperometric measurement for assessing cellular toxicity by monitoring the exocytosis and neurotoxic events in systems get insulted with NMs. Electrochemical impedance spectroscopy (ECIS) is used to track cellular biophysical changes in response to NM interactions. Surface coating and physicochemical electrochemical collision techniques are employed to screen the particle reactivity (Shinde et al., 2020). In regulated settings, electrochemical collision is a newly established approach for fast screening and characterization of particle type, catalytic characteristics, and chemical reactivity. This may help in NPs screening rapidly, without expensive *in vivo* assays. Thus, electrochemical methods are proven to have greater advantages over conventional methods in terms of sensitivity, selectivity, and multiplexing capabilities (Özel et al., 2014).

## Advancements in Predicting the Nanotoxic Effects on Biological Barriers

### BBB

Common cells used in the *in vitro* BBB studies include primary cells like porcine pulmonary artery endothelial cells (BECs) and cell lines like hCMEC/D3, BB19, hBMECs, TY10, and b.End3 cells (Eigenmann et al., 2013). Some investigations have shown that oncogene-transfected hBMECs operate as same as primary cells. As transfected hBMECs have shown good barrier tightness and paracellular permeability, these cell lines are considered promising for establishing an *in vitro* BBB model (Arumugasaamy et al., 2019). Brain endothelial cells derived from iPSCs (induced pluripotent stem cells) and hematopoietic stem cells are also used to develop BBB models (Appelt-Menzel et al., 2017). Fluidics and microfluidics models are other approaches to study BBB function. Microfluidics have become more popular in recent years for more regulated and physiologically appropriate experiments. Microfluidic BBB models will soon replace animal testing to be employed in scientific and clinical research. Advanced microfluidics may represent the future of BBB models due to their design flexibility, capacity to combine coculture methods, and compliance (Arumugasaamy et al., 2019). In addition, novel modeling techniques based on the culture of brain spheroids and organoids were established a few years earlier. The spheroids, which are self-organized dense cellular aggregates grown in low attachment conditions that mimic the 3D environment, also have been modeled in the development of BBB. These spheroids might be used in conjunction with microfluidic devices, such as microvascular networks (Bhalerao et al., 2020). Several computational techniques such as docking, QSAR, and molecular dynamics simulations the paved way for predicting NMs BBB permeation and their potential toxic effects (Shityakov et al., 2017). Novel QSAR and ADMET computational tools and algorithms were also created to assess log BB, PS, and other factors in order to forecast NMs penetration through BBB (Shityakov et al., 2017). CNS organoids, organ-on-chips, spheroids, 3D printed microfluidics, *in silico* models like molecular docking, and other novel technologies implicit the advancements in the field of nanotoxicology (Bhalerao et al., 2020).

### Alveolar Barrier Models for Nanomaterials Toxicity Evaluation

**FIGURE 5:**
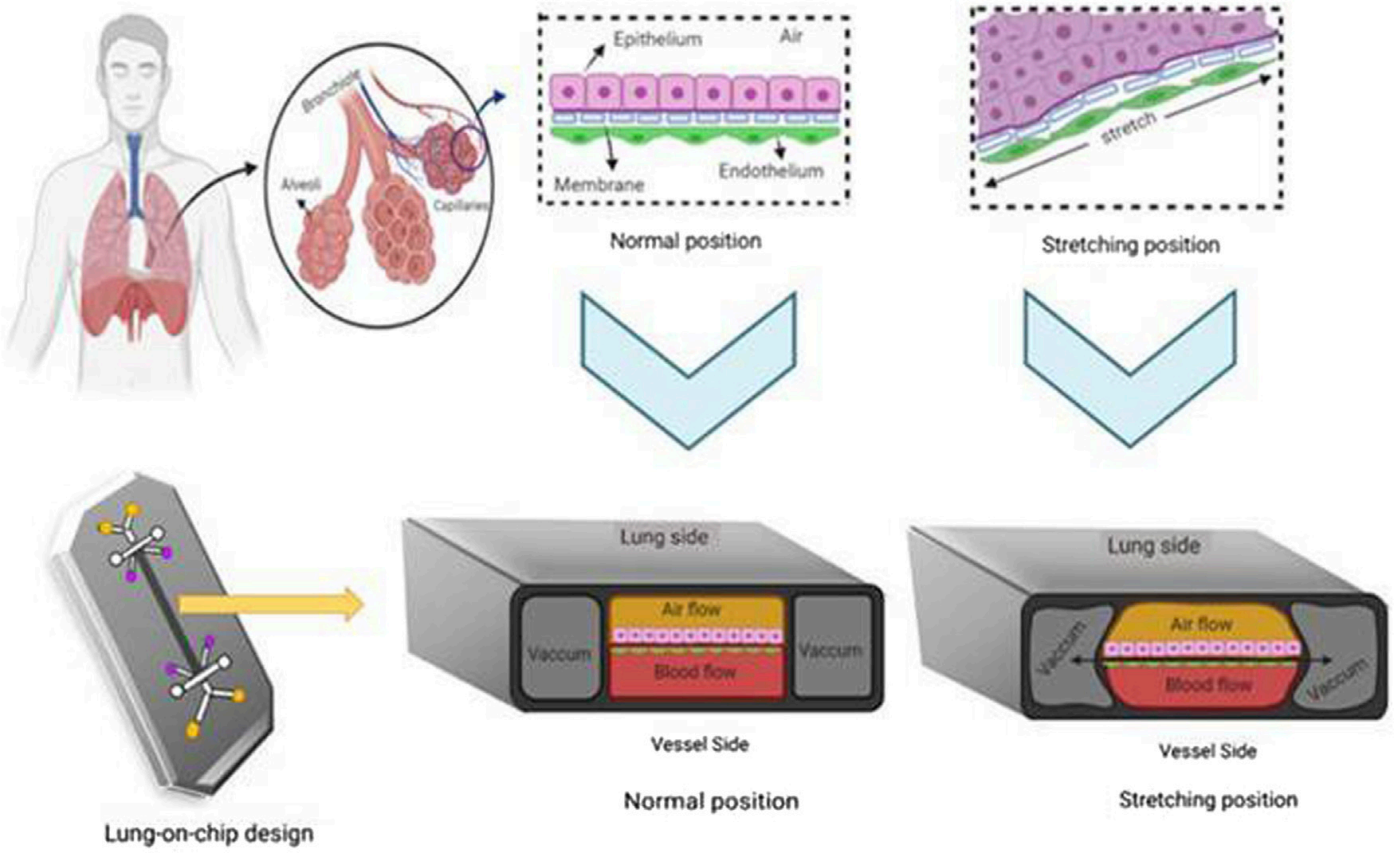
Pictorial representation of the working principle of the organ-on-chip model employed for nanomaterial safety assessment.

As inhalation exposure of airborne substances is unavoidable, exposure of humans to particles is high. Some NMs cross the alveolar barrier and causes pulmonary toxicity. Toxicity testing in *ex vivo* (perfused lungs) models is uncommon because it is only viable for a limited time. In bronchial epithelial cell lines, Calu-3, BEAS-2B, and 16HBE14o cells are frequently employed to test bronchiolar toxicity. Reconstructed bronchial epithelium such as EpiAirway^TM^ is also commercially available which is composed of alveolar epithelial cells and endothelial cells. MRC-5 fibroblasts inculcated in collagen matrix on a Transwell membrane with a top layer covered with the PBMC-derived DCs and 16HBE14o cell lines can be employed for assessing nanotoxicity of NMs (Fröhlich, 2018; Arumugasaamy et al., 2019). Recently, an alternate cell line named human alveolar epithelial lentivirus immortalized (hAELVi) cells are developed which possess good alveolar barrier function (Fröhlich, 2018). In recent years, many coculture models have been established to assess the permeability of NPs through the alveolar barrier. Klein et al. designed a model composed of A459, THP-1 monocytes, and HMC-1 cells seeded on the top part whereas EAhy926 cells are seeded on the basal side of the membrane (Fröhlich, 2018). New approaches have been developed in recent years, like organ-on-a-chip and bioprinted lung models. A study compared the efficiency of manually seeded cells and cell printed models and reported that cell printed models have good barrier integrity than manually seeded cells. Bioprinting might therefore be a more favorable approach for developing future generation lung epithelial models (Figure 5).

### Placental Barrier

Until before the thalidomide tragedy, the placental barrier was an impenetrable barrier between mother and child. Afterward, reports suggested that exposure to many drugs was proven to cause fetal damage. Despite molecules, advents in nanotechnology also produced placental toxicities either intentionally or accidentally. As the placental barrier is the most species-specific mammalian organ, animal experiment data cannot be extrapolated with humans. So, this is the province for the evolution of human-mimicking *in vitro* methods. Conventional methods including Transwell inserts and perfused cotyledon techniques were extensively used for evaluating maternofetal transport. The recirculating dually perfused *ex vivo* human placental perfusion model mimics the mother and fetal blood circulation by perfusing a single cotyledon that is excised from the placenta *ex vivo* (Hougaard et al., 2015). Briefly, at the maternal side of the cotyledon, NPs are added to the perfusate, and measuring perfusate allows the study of particulates through the placenta. Placental toxicity is considered if there is a substantial decrease in NP concentration on the maternal side without an increase in fetal perfusate (Bourget et al., 1995; Grafmüller et al., 2013). This model showed that polystyrene NPs up to 240 nm in diameter could traverse the placental barrier without any obstacle (Wick et al., 2010). Another simplified model, the *in vitro* Transwell^®^ insert, has two liquid compartments separated by a membrane. Cells that mimic the endothelial and epithelial environment can be grown on either side of the membrane. Placental membrane transport can be measured by inserting the substrate on the top of the container and measuring the particle transit in the lower container (Hougaard et al., 2015). Basically, for this model trophoblast cell lines such as BeWo, b30 cloned cells are used to mimic the syncytiotrophoblast (it is a giant multinucleated cell that presents directly in contact with the mother blood, which also provides an endocrine function for placenta) (Correia Carreira et al., 2015). A commendable work initiated by Mathiesen et al. to compare recirculating dually perfused *ex vivo* human placental model and BeWo and B30 cloned monolayer cell transfer model has manifested a high experimental success rate for the BeWo cell transfer model (Correia Carreira et al., 2015). Here, the membrane seeded with trophoblasts exhibited size-dependent transportation as the transport rate was observed to be higher for polystyrene NPs at 50 nm when compared to 100 nm, and similarly for dexamethasone-loaded PLGA, NPs transportation was higher for 146 nm particles in comparison to 232 nm particles (Cartwright et al., 2012; Ali et al., 2013; Mathiesen et al., 2014). But these cell lines might not replicate due to the lack of some cellular transporters (Arumugasaamy et al., 2019). The utilization of primary cells rather than immortalized cell lines to establish primary trophoblast cells can also be used as placental models although they are difficult to grow *in vitro*. The fact that primary trophoblasts derived from the earlier stages of development behave differently than trophoblasts from later stages of development is a primary drawback of this model (Arumugasaamy et al., 2019). Also, newly emerging placenta-on-a-chip models developed with the help of microfluidics and bioengineering in 3D microfabricated devices will definitely expand the era of nanotoxicology (Blundell et al., 2016). In this model, trophoblasts (JEG-3) and Human Umbilical Vein Endothelial Cells (HUVECs) are cultivated on either side of the porous polycarbonate membrane sandwiched between two microfluidic channels (Mosavati et al., 2020). The top and bottom layers of the microfluidic device were fabricated in polydimethylsiloxane (PDMS) using various standard techniques of lithography. A placental chip microdevice was developed and explored for further complicated placental responses to TiO_2_ NPs exposure by Yin et al. (2019). In addition, with these novel methods, the Ussing chamber also mimics the maternofetal transfer as it has two half chambers clamped together with an epithelia sheet of mucosa grown on permeable supports (which isolates the maternal interface from the fetal interface). The chambers are filled with Krebs-ringer buffer to remove all electrical and mechanical driving forces. A freshly prepared placental slice of uniform thickness which is subjected to verification to maintain its placental activity is fixed in the Ussing chamber. It is also found to be a valuable investigating tool to study placental transport (Song et al., 2013). Other approaches include the combination of computational models and the Ussing chamber to evaluate transport. However, in order to construct prediction models that may guide and augment wet-lab studies, these approaches require experimental data.

### Blood-Testis Barrier

NPs spread throughout the body, and signals may transmit between organ systems, ultimately affecting the whole organism. Although crucial organs are safeguarded with their own specific barriers, certain nanosized particles can pass those barriers and cause harm to the human. NPs penetrated through the blood-testis barrier can cause many negative effects on the male reproductive system. Several research attempts were made to recreate an artificial testis by using culture and coculture systems of male germ and Sertoli cells but got in vain. Later developed models are cocultured cells isolated from the rat testis by suspending on a solid support. Legendre and colleagues created an *in vitro* model that replicates the blood-testis barrier. A bicameral chamber of testicular cells (peritubular, Sertoli, and germ cells) obtained from 18-day-old rats constitutes this 3D-engineered Blood-Testis Barrier (eBTB). It could be a promising alternative approach to animal reproductive toxicity studies (Legendre et al., 2010). And impedance-based measurements are also used to estimate the blood-testes barrier damage. There are several impedance devices on the market that enable sensitive real-time monitoring of cell changes (Drasler et al., 2017).

### Reproductive Toxicity

Nowadays, continuous exposure to NPs has been added as a threat to the vulnerable reproductive system. When compared to males, the female reproductive system is more sensitive mainly owed to a limited number of gametes production in their life cycle (Sun et al., 2013). After exposure, through circulation NPs reach the female reproductive system and invade protective barriers such as theca cells, granulosa layers, and zona pellucida, which are mainly involved in the protection of oocyte during the oocyte maturation and exhibit apoptosis and antrum formation by accumulating in the ovarian cells. There is a shred of clear proof that oxidative stress and inflammation might lead to reproductive and developmental toxicity. Upon exposure, NPs are taken up by cells present in the placenta, which has several Toll-like receptors (TLR-2 and TLR-4) on their surface inducing various inflammatory responses. Some NPs alter the expression of gene encoding proteins involved in the synthesis of gonadotropin-releasing hormone (GnRH), follicle-stimulating hormone (FSH), and luteinizing hormone (LH) which aids in releasing ovarian sex hormones such as estrogen and progesterone through direct alteration in secretory cells and corpus luteum or by indirect alteration in the hypothalamus-pituitary ovarian axis (HPO axis) by crossing BBB (Yamashita et al., 2011). Long-term exposure of TiO2 NPs (10 mg/kg) in female nonpregnant mice resulted in increased estradiol synthesis by upregulation of CYP17a1 gene and also upregulated 18 genes and downregulated three genes related to apoptosis. Alterations in sex steroid levels, increased apoptosis, ROS generation, and ovaries inflammation cumulatively cause damage to ovaries and female infertility (Busquet et al., 2008). In the view of Wick et al., the NPs of size less than 240 nm are most likely to get through the placental barrier. This NPs transplacental passage causes development toxicity by causing deleterious effects to fetal development. NPs exhibit their toxicity on embryo/fetal tissue by directly reaching those tissues or by impacting maternal organisms which influence and alter the genes associated in the production of ROS, inflammation, or apoptosis. In a reported study, intravenous (IV) administration of high dose SiO2 and TiO2 NPs for 2 days consecutively reduced uterine weight and produced smaller fetuses in pregnant mice. ROS and inflammation also induce effects not only in reproductive toxicity but also in developmental toxicity. In another study, intratracheal administration of higher dose CNTs caused fetal abnormalities along with a significant increase in WBC count in maternal blood. But on the other side, at lower doses of CNTs, producing neither malformations nor WBC increase was reported (Ema et al., 2016).

In the male reproductive system, NPs affect the HPG axis and increases ROS levels which result in decreased spermatogenesis. NPs can enter sperm and pause acrosome reaction and its motility by attaching to the mitochondria present in the head and tail parts of sperm (Boisen et al., 2013). NPs at the hormonal level alter testosterone, FSH, and LH levels and cause DNA damage and fragmentation. At the cellular level, NPs affect the quality and quantity of sperm and Leydig cells. At the organ level, NPs make histological alterations, change the structure of the reproductive organ, cause damage to testes, decrease epididymis and testes weight, empty seminiferous tubules, and alter seminiferous tubules diameter and morphology (Iftikhar et al., 2021). Besides male and female reproductive system and transplacental barrier, they are also involved in altering fetal growth and organ formation by causing teratogenic effects (reducing bone, sternum, toes, and fingers formation) and mortality. Nevertheless, NPs were also reported to interact with lactation in feeding mothers (Brohi et al., 2017).

Reproductive and developmental toxicity accounts for more than 20% of the preclinical-related toxicity attrition in the drug development pipeline. In process of evaluation of reproductive toxicity of NPs, analyzing germ cells provides an excellent opportunity to examine the nanotoxicity. Spermatogonial stem cells obtained from the male reproductive system can act as the best *in vitro* model to compare the nanotoxicity of various NPs (Braydich-Stolle et al., 2005). Another approach called impedance-based measurements is used to estimate the blood-testes barrier damage. There are several impedance devices on the market that enable sensitive real-time monitoring of cell changes (Drasler et al., 2017). Various *in vitro* coculture models like Sertoli-germ cell cocultures, Sertoli cell-enriched cultures, germ cell-enriched cultures, Leydig cells, Leydig cell-Sertoli cell cultures, peritubular cells, and tubular cultures are available today to assess testicular toxicology (Gray, 1986; Lamb and Chapin, 1993). A recently designed testicular cell culture dual-compartment model can somewhat replicate the typical physiological activities. In this model, isolated mammalian Sertoli cells from mammalian testes are cultured on a Millipore filter that made solid support between the two fluid compartments, which creates an epithelial layer with tight junctions and high polarization that obstructs the nanotoxic compounds through the gap between two compartments same as like blood-testis barrier (Steinberger and Klinefelter, 1993). In another study, by using two-compartment models made of Sertoli cell monolayers, different dose effects (0.75–24 µM) of cadmium chloride (CdCl2) on transepithelial electrical resistance are observed. This method and experimental model will be highly valuable in toxicological examinations of the blood-testis barrier, particularly those that are suspected of compromising the barrier’s integrity. This model can also be implemented in nanotoxicity evaluation (Janecki et al., 1991; Janecki et al., 1992). Besides, novel cytotoxicity assays, omics technologies, microfluidics, stem cells, etc., can also be implemented in the assessment of testicular toxicology. *In silico* computational techniques have aroused a lot of interest in the field of nanotoxicology these days. Molecular docking is the best structural-based approach which explains molecular interactions of NPs with several molecules. The NPs produce negative effects in the body by generating oxidative stress and these molecular interactions can be studied finely by molecular docking. Verma et al. explored the interaction of ZnO NPs with a protein called sod, a well-known oxidative stress regulator (Hougaard et al., 2015). Computational methods were developed targeting kinases in Sertoli cells, which are involved primarily in spermatogenesis such as assembling and dissembling of blood-testes barrier and apical ectoplasmic specializations. GROMACS molecular dynamics and PyMOL viewer have been used to study molecular dynamics simulation and structural analysis, respectively (Jenardhanan et al., 2018).

With great advancements in microfluidics and organ-on-chips research in the last decade, several researchers have developed 3D engineered devices that replicate various organs of the female reproductive system which includes modeling of endometrium, placenta, and uterus. More recent works manifested the possibility of reproducing the whole menstrual cycle by connecting all the possible tissues on a microfluidic device. Cocultured cell types used in placenta-on-chip, uterus-on-chip, and endometrium-on-chip are human trophoblast (BeWo b30/JEG-3) cell lines, mouse oocyte, and human primary endometrial stromal cells, respectively. Uterus-on-chip was created to address the shortcomings of *in vitro* fertilization-embryo transplantation (IVF-ET) by simulating uterine processes such ovulation, embryo growth, and insemination. Briefly, it contains two PDMS layers; the upper layer is shaped as a zig-zag channel to allow the attachment of the oocyte (Wei-Xuan et al., 2013). Gnecco et al. created an endometrium-on-chip mimicking humans, which can be useful to visualize mutual interactions (Mancini and Pensabene, 2019). In 2013, Woodruff created the first organ-on-chip recreating a 28-day menstrual cycle, where the human cells from several reproductive organs are grown in a network of microengineered units (Xiao et al., 2017). Rat preantral follicle *in vitro* culture systems can be applied to reproductive biology and toxicology research (Sun et al., 2004; Xuying et al., 2011). The CALUX battery tests are a set of 24 molecular assays that may be used to track changes in the activity of major transcription factors ranging from nuclear receptors to transcriptional factors involved in cellular communication (Piersma et al., 2013).

### Developmental Toxicity

In the assessment of developmental toxicity, whole rat embryo culture (WEC) is frequently utilized technique for many years. It permits the intact culture of early-stage embryos in their visceral yolk for almost up to 3 days. This phase is sensitive to teratogenic insults and also covers almost all the morphogenic processes such as neurulation, limb bud formation, facial morphogenesis, and cardiac looping which occur during the 1st trimester of human pregnancy (Zhang et al., 2016). Although harvesting and preparation of rodent embryos for culture need specialized trainers, it is difficult to remove extra embryonic membranes without losing the integrity of the embryo. But the stumbling block is that this method does not reflect the effects shown by maternal metabolism opening the door to find newer toxicity assays (Augustine‐Rauch et al., 2010; Van Der Laan et al., 2012). The second model, zebrafish embryo culture, is found to have some special advantages towards P450 and various CYP activities that other developmental assays (Van Der Laan et al., 2012). But the limiting factor is that the liver will be in unfunctional state till day four after fertilization, indicating the incomplete metabolism mainly during the teratogenic sensitive window, i.e., organogenesis (Zhang et al., 2016). So, to fulfill the gaps, researchers have expanded this method by combining zebrafish embryonic cultures and mammalian hepatic microsomes. In order to assess the teratogenic potential, microsome-produced metabolites were given to embryos (Busquet et al., 2008). Pressure to reduce animal models by obeying the 3Rs concept while testing pharmaceuticals gave booming rise to start ESC research. As discussed in the previous section *Stem Cells as a Novel Approach to Predict Toxicity*, the most widely used model to screen is the human or mouse ESC. This method consists of a group of ES cells that could be able to aggregate as embryoids and differentiate into cardiomyocytes (Augustine-Rauch et al., 2010). A newly emerged ReProGlo assay used mouse embryonic cells (mES) transfected with Wnt-responsive luciferase reporter vector to assess the changes in the canonical Wnt/β-catenin signaling pathway, a central and crucial pathway in early embryonic development (Uibel et al., 2010; Piersma et al., 2013; Uibel and Schwarz, 2015). Through the comparison results of mouse and human ESCs, it is proved that most of the miRNAs present in humans are not actively expressed in mouse which led the researchers to explore human ES cells more. A fair alternative approach to hESC is human-induced pluripotent stem cells (hIPS). hIPS and hESCs face vast differences in terms of gene and microRNA expression (Zhang et al., 2016). However, some challenges are still required to be concluded with supportive research to implement hIPS as potential replacements in embryotoxicity and teratogenicity testing.

## Methods to Predict Biodistribution of Nanoparticles

Today, various analytical imaging techniques are available including Laser confocal microscopy (LCM), laser ablation inductively coupled plasma mass spectrometry (LA-ICP-MS), transmission electron microscopy electron energy loss spectroscopy (TEM-EELS), and transmission electron microscopy energy dispersive X-ray analysis (TEM-EDX) which are mainly used to evaluate the distribution and chemical moieties of NPs in the biological samples. In addition, synchrotron radiation microbeam techniques like SRXRF (synchrotron radiation X-ray fluorescence) and SRXAS analysis are used in mapping biodistributions and to identify the chemical status of NPs both in *in vivo* and *in vitro* models (Al Faraj et al., 2009; Elci et al., 2016).

## Adverse Outcome Pathways in the Assessment of Nanomaterial Toxicity

Over the last 2 decades, nanotechnology emerged as the most rapidly growing application in various sectors of scientific research throughout the world. Over time, application of NMs has been increased in various fields such as automotive, biomedical, pharmaceutical, defense, and electronics. Increased application and usage lead to increased demand in the production of NMs; by 2021, it is estimated that business could reach up to $90.5 billion (Sengul and Asmatulu, 2020). Increased use of NMs could also raise a concern about safety and underlying risk to humans and the environment. Currently, the various possible biological effects and toxicity of NM are studies in various laboratories throughout the world; however, testing strategies for current and newly produced NM could take years for validation and investigation. To minimize toxicity testing and efficient use of their existing molecular data, there was a need to develop a conceptual framework to predict toxicity outcomes. In 2012, the OECD, together with the EU-Commission’s Joint Research Centre, US-EPA, and US Army Engineer Research and Development Center, launched a new initiative to share and discuss the development of adverse outcome pathway (AOP)-related knowledge. To develop AOP knowledge, the initiative brings four different platforms (AOP-Wiki, Effectopedia, Intermediate Effects DB, and AOP Xplorer) to collaborate and facilitate the sharing of AOP-related knowledge between scientists and stakeholders throughout the world. AOP is a conceptual framework linking a perturbation at the molecular level of a biological system with an adverse (apical) outcome at higher levels of biological organization, which are of regulatory relevance (e.g., impact on growth, reproduction, or survival).

**FIGURE 6:**
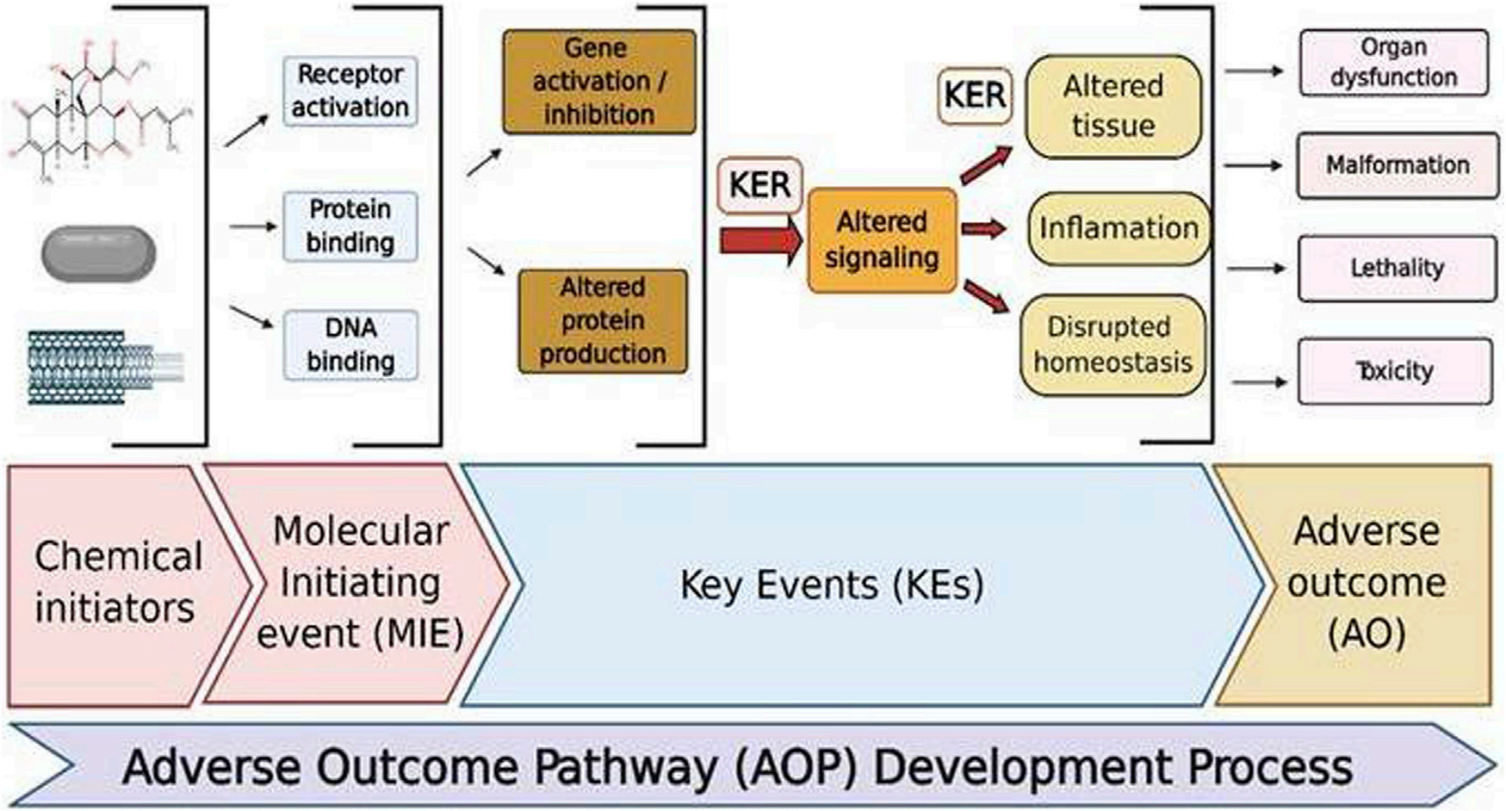
Process of development of AOP includes chemical initiators (chemical or NPs or nanotubes) which will bind to receptor, protein, or DNA causing cascade impact on the signaling pathway. The initial interaction with biological system is the molecular initiating event (MIE), which further leads to the development of key events (KEs) and finally causes apical adverse outcome (AO). The relationship between the two key events is designated as key event relationships (KER).

The structure of AOPs includes the description of key events (KEs) which are xenobiotic induced responses at the molecular, cellular, organ, or suborganismal levels which are measurable and required for an AO to occur. The initial KE represents the molecular initiating event (MIE), whereas the last KE represents the AO. The MIE is the primary site of interaction between a chemical stressor and its molecular target within an organism. Based on the literature, these molecular interactions can be either highly specific, such as binding to a specific receptor, or nonspecific, such as a reactive chemical binding to various cellular proteins leading to toxicity. Events that lead to changes in the cell state, metabolic pathways, signal transduction, or cell function from the MIE pass via a sequence of KEs. Finally, all these processes lead to an AO that is the traditional apical endpoint for the risk evaluation of chemical substances. In the case of NPs, specific binding to proteins is rarely observed; most of the time NP induces toxicity through nonspecific mechanisms (see Figure 6). Several studies have used the AOP framework for chemical risk assessment since the inception of the AOP concept, because of its promising qualities. An analysis of the mechanisms linking a molecular event to an apical endpoint based on KEs, which eventually reduces toxicity testing or guides research in order to address knowledge gaps, was the main aim of the AOP idea. As of now, a large portion of the AOPs is significantly centered around the chemical-induced toxicity outcome pathways. However, there is a significant interest provided to investigate the AO caused by various forms of chemicals such as NMs and particles. Till now, the research outcomes suggest that AOPs made for general chemicals applicable for the NMs which are made with the same chemicals; however, details of understanding of MIE is not done and yet which need to be further investigated; however, details of understanding of MIE are not done yet which need to be further investigated. Also, NM toxicity is influenced by its size as the size can influence the physicochemical properties of NMs which results in unique biological interaction which eventually resulted in enhanced toxicity outcomes. In addition to chemicals, NMs toxicity in biological systems is unique because of their surface properties which will govern interaction with biological systems which can lead to a highly crucial cellular uptake and internalization for NM-induced toxicity could serve as MIE for NM-induced AO. Unlike chemicals, NMs biological interaction could be initiated in various ways including mechanical, physical, chemical, and receptor-mediated, and NM could initiate multiple outcomes with specific and no specific interactions. As discussed earlier, NMs toxicity and AO is usually followed as chemicals by which the NMs are made; however, due to additional properties, MIEs triggering for NMs is very vague and not yet understood completely, which further creates a knowledge gap to investigate further for NM-mediated understanding for MIE and leading KEs and finally AO.

To complete the knowledge gaps in AOP development, the Comparative Toxicogenomic Database will provide curated chemical/(nano)particle, genes, and disease connections, where genomic and molecular profiles can be analyzed for association with disease progression. Only a number of current researches have recently focused on MWCNTs exposed workers, when their blood samples were analyzed, genomic markers for various pathways, pulmonary, and cardiovascular-related molecular processes (Shvedova et al., 2016). Further, mapping their interpreting mechanisms about the development of lung diseases can be easily done using the AOP. Currently, various AOPs concerning MWCNTs in the progression of lung associate AO are being developed through collaborative efforts including various researchers around the globe which are “Secretion of inflammatory cytokines leading to lung fibrosis” (AOP:173) (Labib et al., 2015), “EGFR [Epidermal Growth Factor Receptor] Activation Leading to Decreased Lung Function” (AOP:148), and “Chronic cytotoxicity of the serous membrane leading to pleural/peritoneal mesotheliomas in the rat” (AOP:171). In addition, using bioinformatics tools will also facilitate the linkage between AOPs and KEs. These analyses are utilized to create effective models for illness prediction assessment techniques and biomarkers identification and to better comprehend various conditions of disease (Nymark et al., 2018).

**FIGURE 7:**
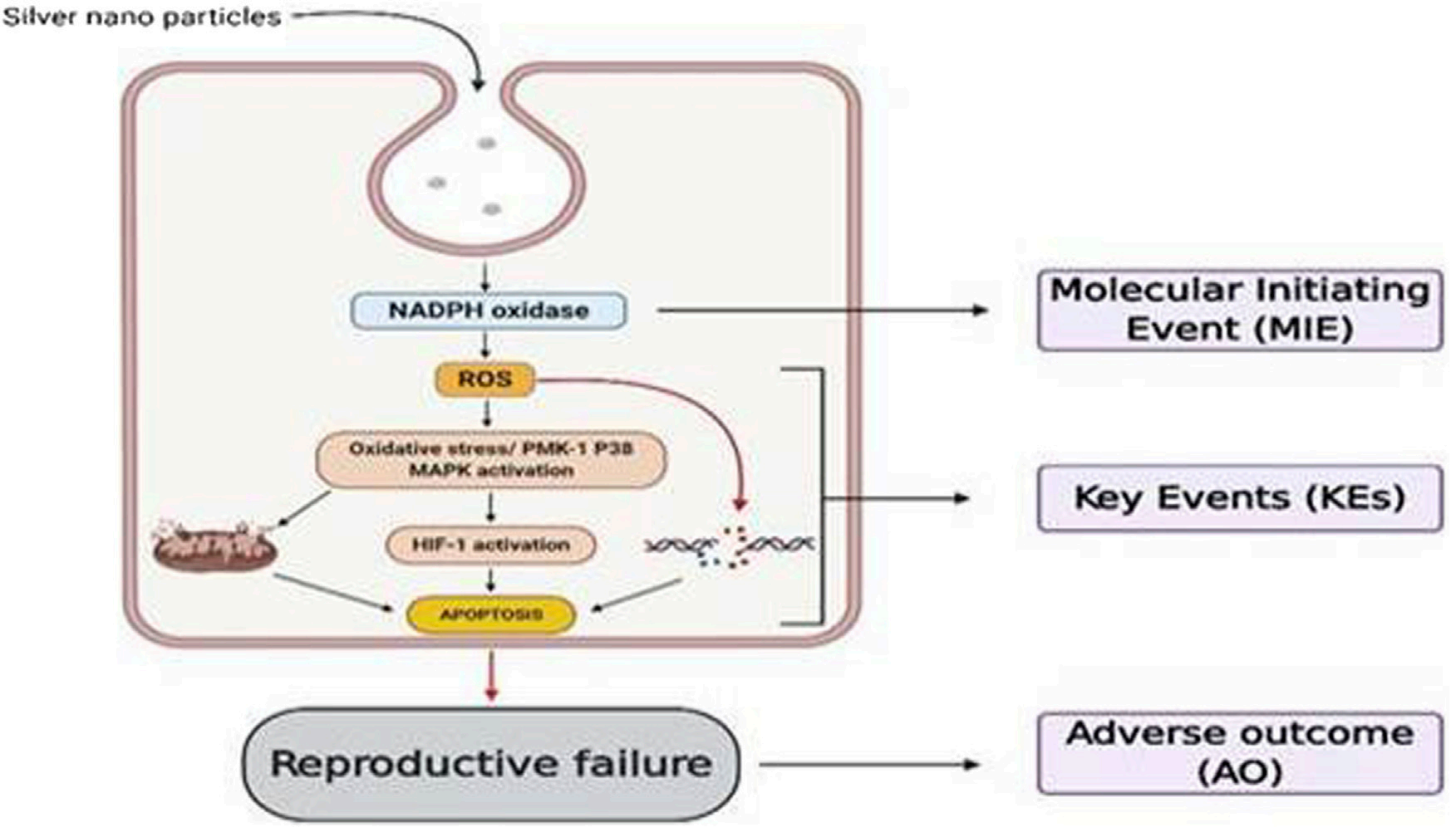
Illustration of the mechanistic pathway by which AgNPs will cause reproductive toxicity in *C. elegans*.

In recent years, Jeong et al. (2018) investigated the underlying molecular pathways caused by silver NPs (AgNPs) in *C. elegans*. Using this study, the groups have developed an AOP using transcriptomics, molecular pathways, and biochemical tools. Study results suggested that oxidative stress is a major MIE for the responsible reproductive toxicity caused by AgNPs. The groups have conducted various experiments to establish a relationship between MIE and AO, which includes NADPH oxidase, ROS formation, PMK-1 P38 MAPK activation, HIF-1 activation, mitochondrial damage, DNA damage, and apoptosis (see Figure 7). The building of these KEs based on experimental evidence provided concrete evidence of a causal relationship between MIE and AO (Jeong et al., 2018).

A study reported by Ma et al. (2018) examined the potential toxicity mechanisms of AgNPs in aquatic species using a zebrafish model. This study investigated possible mechanisms involved in causing reproductive damage and related potential AOP in zebrafish for 5 weeks at various concentrations following chronic exposure to AgNPs (0, 10, 33, and 100 mg/L). AgNPs exposure indicated much lower fecundity in female zebrafish, which was further supported by increased apoptotic cells in ovarian and testicular tissues using TUNEL testing. Increased accumulation of silver NPs in tissues leads to increased ROS production which was also found in both ovary and testis. ROS-induced apoptosis was further validated by analyzing the transcription levels of various genes (Bax, bcl-2, caspase-3, and caspase-9) associated with the mitochondrion-mediated apoptosis pathway. In conclusion, research indicates that exposure to AgNPs produced oxidative stress and triggered death in germ cells via the mitochondrial-dependent pathway, ultimately leading to the impaired reproductive potential of zebrafish (Ma et al., 2018b).

Overall, both studies mentioned above will be a great example of how the AOP can be developed using current data and undertake more trials to bridge the gaps between MIE, KE, and AO. Currently, so many researchers are working on the development of AOP with respect to NP-induced toxicity on various models; AOPs which are currently in various stages of development are listed in Table 3.

**TABLE 3:** List of AOPs which are currently in various stages of development focused on NPs

S. no.	Stressor	AOP number	AOP title	MIE	KEs	Adverse outcome	Status
1	NPs	AOP: 144	Endocytic lysosomal uptake leading to liver fibrosis	Endocytotic lysosomal uptake	1) Lysosome disruption	Liver fibrosis	Under review
					2) Mitochondrial dysfunction 1		
					3) Cell injury/death		
					4) Increased proinflammatory mediators		
					5) Leukocyte recruitment/activation		
					6) Stellate cells activation		
					7) Collagen accumulation		
2	Silver NPs	AOP: 207	NADPH oxidase and P38 MAPK activation leading to reproductive failure in *Caenorhabditis elegans*	NADPH oxidase activation	1) ROS formation	Reproductive failure	Under development
					2) Increase, oxidative stress/activation, and PMK-1 P38 MAPK		
					3) HIF-1 activation		
					4) Increased DNA damage repair		
					5) Mitochondria damage		
					6) Apoptosis		
3	UV-activated titanium dioxide NPs	AOP: 208	Janus kinase (JAK)/signal transducer and activator of transcription (STAT) and transforming growth factor- (TGF-) beta pathways activation leading to reproductive failure	Under investigation	1) JAK/STAT pathway activation	Reproductive failure	Under development
					2) TGF-beta pathway activation		
4	Silica NPs	AOP: 209	Perturbation of cholesterol and glutathione homeostasis leading to hepatotoxicity: integrated multi-OMICS approach for building AOP	Under investigation	1) SREBF2 upregulation	Hepatotoxicity	Under development
					2) Unsaturated fatty acid upregulation		
					3) GSS and GSTs gene downregulation		
					4) Glutathione synthesis		
					5) 3-Hydroxy-3-methylglutaryl-CoA reductase gene activation		
					6) Perturbation of cholesterol		
					7) Glutathione homeostasis		
5	Graphene oxide NPs	AOP: 210	Activation of c-Jun N-terminal kinase (JNK) and forkhead box O (FOXO) and reduction of Wnt pathways leading to reproductive failure: integrated multi-OMICS approach for AOP building	Under investigation	1) Peptide oxidation	Reproductive failure	Under development
					2) JNK activation		
					3) FOXO activation		
					4) Wnt pathway inhibition		
					5) Defect of embryogenesis		
6	Carbon nanotubes	AOP: 241	Latent TGF-beta 1 activation leads to pulmonary fibrosis	TGF-beta 1 activation	1) Increase in the differentiation of fibroblasts	Pulmonary fibrosis	Under development
					2) Induction of epithelial-mesenchymal transition (EMT)		
					3) Collagen accumulation		
					4) TGF-beta pathway activation		
7	Carbon nanotubes, multiwalled carbon nanotubes, single-walled carbon nanotubes, and carbon nanofibres	AOP: 173	Substance interaction with the lung resident cell membrane components leading to lung fibrosis	Interaction with the lung resident cell membrane components	1) Increase in the secretion of proinflammatory and profibrotic mediators	Pulmonary fibrosis	Under review
					2) Increased recruitment of inflammatory cells		
					3) Loss of alveolar-capillary membrane integrity		
					4) Increased activation of T (T) helper (h) type 2 cells		
					5) Increased fibroblast proliferation and myofibroblast differentiation		
					6) Increased extracellular matrix deposition		
8	Insoluble nanosized particles	AOP: 237	Secretion of inflammatory cytokines after cellular sensing of the stressor leading to plaque progression	Sensing of the stressor by pulmonary cells	1) Increased production of pulmonary and proinflammatory cytokines	Plaque progression in arteries	Under development
					2) Increased production of pulmonary SAA		
					3) Formation of HDL-SAA		
					4) Increased systemic total cholesterol pool		
					5) Foam cell formation		

As discussed above, major challenges in the development of NM safety evaluation are due to the lack of quality scientific data. Due to rapid development in nanotechnology, new developments of novel nanomaterial are growing rapidly because of their widespread usage in industries. However, to complete toxicity testing using animal studies for complete safety assessment would take many years and will consume billions of dollars. At present day, various *in silico* and *in vitro* methods are playing a major role as an alternative approach of *in vivo* models (Lilienblum et al., 2008). These methods being used in safety assessment incorporation of AOP framework will help in the effective use and interpretation of data generated and help in direct correlation to the human population. The application of AOP in research and regulatory decision-making is completely relayed on the accuracy of the biological mechanisms reported, the biological plausibility of KEs, and their measurability, finally supporting the weight of the evidence presented to support KERs (Gerloff et al., 2017; Ede et al., 2020). A criterion needs to be incorporated while selecting the biomarkers from a vast number of pleiotropic and redundant molecular pathways should be carefully established while the investigator is planning for testing. Those studies will help in building AOP to be more robust and minimize the toxicity of chemical causes toxicity in similar pathways (Halappanavar et al., 2019).

## Conclusion

Nanotechnology is a swiftly evolving field involved in inventing and developing nanoscale range materials and devices with the collaboration of multiple disciplines (Gorman et al., 2004; Schummer, 2004; Porter and Youtie, 2009). These inventions had shown a great revolution in the fields of agriculture, medicine, and electronics with its widespread global business which is expected to reach more than 100 billion US dollars by the end of 2024 (Matteucci et al., 2018). It is quite common that, in the process of developing new technologies especially for those intended to improve human health, there exist risks along with addressing the concerns. On common ground, the easiest yet complicated way of avoiding the undesired immune complications is to understand the material behavior and interactions and predict the physiological response upon subjecting to the human (Schaeublin et al., 2014; Beddoes et al., 2015; Zhou, 2015). To date, numerous studies were performed to assess the safety of NMs but the space between the *in vitro* and *in vivo* outcomes and clinical observations exists even today (Yadav et al., 2013). To reduce this gap, the mechanistic events involved in producing the toxicity should be thoroughly studied, and advanced techniques need to be geared up to overcome present-day conventional method difficulties.

Here, in this review, we initially discussed the widely applied NMs employed in biomedicine followed by the most common mechanisms of toxicities associated with the NMs. As the conventional method of risk, identification is narrowed today with their minimal and vague results in nanotoxicity evaluation and thus the conventional methods that are routinely practiced in the laboratories were discussed and the flaws in the current utility were highlighted. To narrow the gap between preclinical and clinical observations, several novel and advanced methods for evaluating the nanotoxicity of the NMs were developed which are in the budding stages of development. The advent of novel technologies with the novel application has gained immense interest in the nanotoxicology field with the integration of multidisciplinary areas. This greatly intensifies the safety concerns which are lacking with conventional methods due to their unspecific and reliable data. To overcome advanced methods to overcome these concerns is highly recommended for hassle-free development and translation of NMs in nanomedicine. In this regard, we have explored the newly evolving techniques and reported them in the current review. These techniques demonstrated the advancement in identifying the probability of even minute toxicological effects which will aid the research communities in developing safe nanomedicines for human use.

However, apart from the identified toxicities, through our literature survey, we identified that, during nanomedicine development, some factors that are considered minimal are usually underrated which are highly impacting nanotoxicology today. These include the following: 1) While designing the nanomedicine, more focus is made on the cargo that is enclosed by the NPs while the least concentration is made on the NMs due to which, toxicity concerns are arising with their long-term exposure (Wang et al., 2015b; Wu et al., 2015). 2) Reporting and publication: once obtained with any nanotoxicity observation, mild to minimal toxicities are least reported (Donaldson and Poland, 2013). This should be avoided with transparent reporting which can avoid further developmental experiments and expenses as well. 3) Also, the potential effects of NPs on risk factors should prioritize with utmost care while designing NPs. These include pregnant females, geriatrics, pediatrics, neonates, and patients with comorbid conditions (Armstead and Li, 2016). For instance, maternal exposure to NPs highly develops the inflammatory cytokines that exhibit high affinity to reach the fetus and induce alterations in gene expression, and thereby damage of DNA is seen. In comorbid patients, NPs may induce the inflammation with their entry and may aggravate other disease conditions by their systemic circulation and exaggerate the oxidative stress and inflammation more specifically in cardiac and pulmonary disease patients. When we compare the cost of conventional medicine and nanomedicine, the cost of nanomedicine is high because of the increased specifications to evaluate different toxicities that may arise with it (Faunce and Shats, 2007; Bosetti and Jones, 2019). It is also essential to undergo various panels of tests to limit the additional health risks with target activity. We conclude that integrating multidisciplinary in nanotoxicology with initial risk assessment can minimize the risk of NPs in producing nanotoxicology. Taken together, nanotoxicology is a challenging field to identify, understand, and resolve the unpredicted effects due to their complexity. Hence, it is necessary to be circumventing the consequences from initial development with the aid of interdisciplinary as there is no single method to meet all the crucial requirements as observed from the present study.
